# High altitude hunting, climate change, and pastoral resilience in eastern Eurasia

**DOI:** 10.1038/s41598-021-93765-w

**Published:** 2021-07-12

**Authors:** William Taylor, Isaac Hart, Caleb Pan, Jamsranjav Bayarsaikhan, James Murdoch, Gino Caspari, Michael Klinge, Kristen Pearson, Umirbyek Bikhumar, Svetlana Shnaider, Aida Abdykanova, Peter Bittner, Muhammad Zahir, Nicholas Jarman, Mark Williams, Devin Pettigrew, Michael Petraglia, Craig Lee, E. James Dixon, Nicole Boivin

**Affiliations:** 1 Department of Archaeology, Max Planck Institute for the Science of Human History, Jena, Germany; 2 Department of Anthropology, University of Colorado-Boulder, Boulder, CO USA; 3 Department of Anthropology, University of Utah, Salt Lake City, UT USA; 4 Innov8.ag Solutions, Walla Walla, WA USA; 5 National Museum of Mongolia, Ulaanbaatar, Mongolia; 6 Wildlife and Fisheries Biology Program, Rubenstein School of Environment and Natural Resources, University of Vermont, Burlington, VT USA; 7 Institute for Archaeological Science, University of Bern, Bern, Switzerland; 8 Department of Archaeology, Sydney University, Sydney, Australia; 9 Institute of Geography, University of Göttingen, Goldschmidtstr. 5, 37077 Göttingen, Germany; 10 Harvard University, Cambridge, MA USA; 11 Preservation Management Office for the Mongolian Altai Rock Art Complex, Bayan-Ulgii, Mongolia; 12 ArchaeoZOOlogy in Siberia and Central Asia - ZooSCAn, CNRS – Institute of Archaeology and Ethnography SB RAS International Research Laboratory, IRL, 2013 Novosibirsk, Russia; 13 American University of Central Asia, Naryn, Kyrgyzstan; 14 University of California-Berkeley, Berkeley, CA USA; 15 Department of Archaeology, Hazara University, Mansehra, Pakistan; 16 Valles Caldera National Preserve, U.S. National Park Service, Jemez Springs, NM USA; 17 Department of Anthropology, University of New Mexico, Albuquerque, NM USA; 18 SWCA Environmental Consultants, Albuquerque, NM USA; 19 School of Social Science, University of Queensland, Brisbane, Australia; 20 Department of Anthropology and Archaeology, University of Calgary, Calgary, Canada; 21 Department of Anthropology, National Museum of Natural History, Smithsonian Institution, Washington, D.C USA; 22 Institute of Arctic and Alpine Research (INSTAAR), University of Colorado at Boulder, Boulder, CO USA

**Keywords:** Archaeology, Climate-change impacts

## Abstract

The transition from hunting to herding transformed the cold, arid steppes of Mongolia and Eastern Eurasia into a key social and economic center of the ancient world, but a fragmentary archaeological record limits our understanding of the subsistence base for early pastoral societies in this key region. Organic material preserved in high mountain ice provides rare snapshots into the use of alpine and high altitude zones, which played a central role in the emergence of East Asian pastoralism. Here, we present the results of the first archaeological survey of melting ice margins in the Altai Mountains of western Mongolia, revealing a near-continuous record of more than 3500 years of human activity. Osteology, radiocarbon dating, and collagen fingerprinting analysis of wooden projectiles, animal bone, and other artifacts indicate that big-game hunting and exploitation of alpine ice played a significant role during the emergence of mobile pastoralism in the Altai, and remained a core element of pastoral adaptation into the modern era. Extensive ice melting and loss of wildlife in the study area over recent decades, driven by a warming climate, poaching, and poorly regulated hunting, presents an urgent threat to the future viability of herding lifeways and the archaeological record of hunting in montane zones.

## Introduction

Artifacts emerging from melting ice provide a rare archaeological dataset to understanding the emergence of pastoral economies in the frigid steppes of interior Eurasia. Across the globe, the survey of permanent patches of snow and ice has produced new insights into the prehistoric use of high-elevation or high latitude regions which suffer from poor representation in the archaeological record^1^. Glaciers and ice sheets serve as important travel corridors across high mountains^2,3^, and in summer, the melting ice from both glaciers and “ice patches” (non-glacial snow and ice accumulations which in the Northern Hemisphere often occur on protected north-facing slopes) provides an important source of freshwater used by animals, plants, and humans. In many areas of the world, such features function as a hunting hotspot for larger mammals, which congregate seasonally on the ice for various reasons such as thermoregulation, hydration, escape from insect pressure, and improved forage^4,5^. When organic artifacts or biological material becomes incorporated in these features, they can be preserved intact, providing a rare snapshot into cultural activity for this otherwise high-energy depositional environment that rarely preserves organic archaeological material*.* Due to global climate change, ancient cultural and biological material preserved in the ice for centuries or millennia is now melting out for the very first time.

### Mountain zones and the emergence of herding economies in Eastern Eurasia

**Figure 1:**
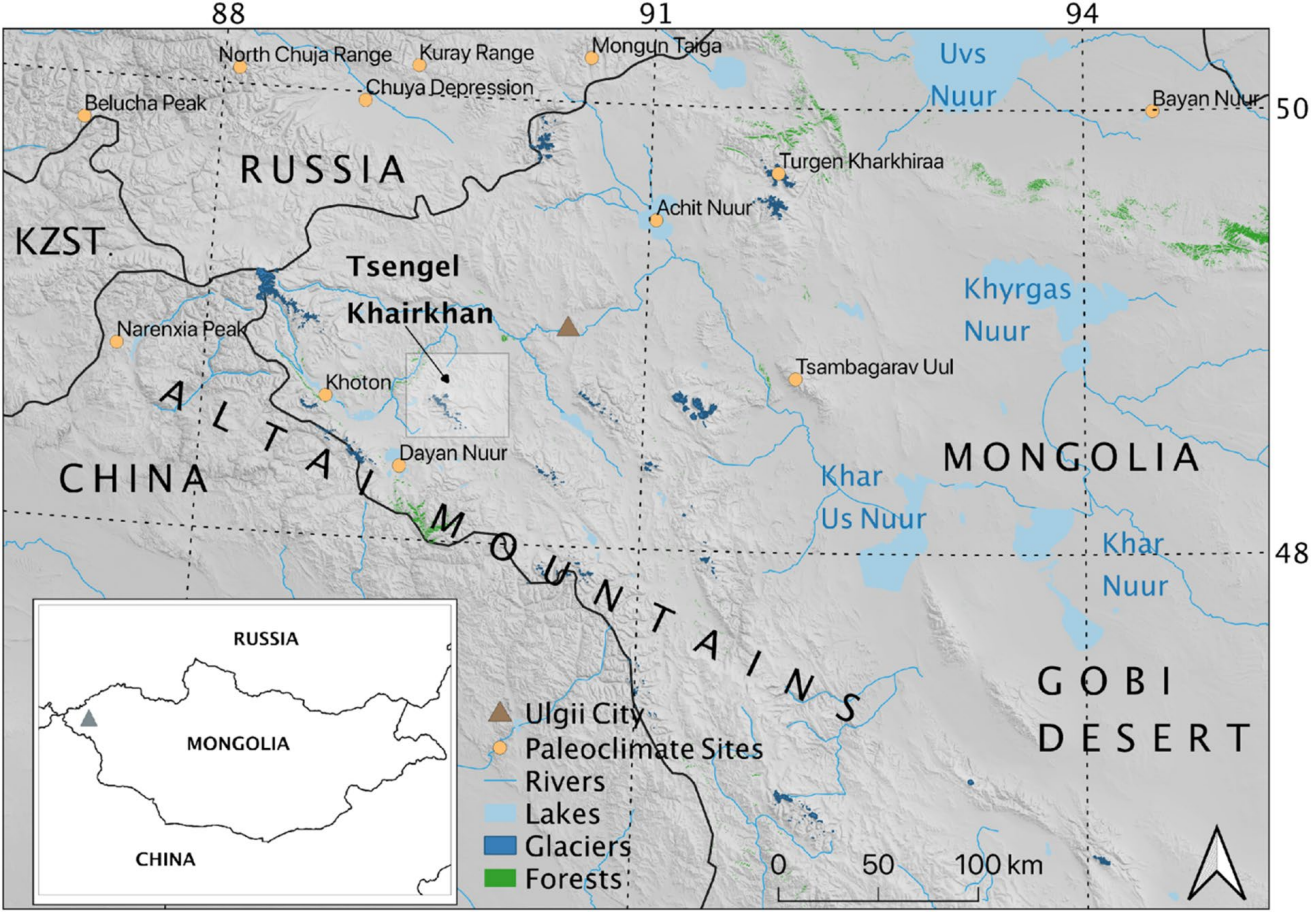
Location of the Tsengel Khairkhan study site and key geographic locations mentioned in the text (including paleoclimate study sites referenced in the Supplementary Material). Produced in QGIS 3.10 (http://www.qgis.org).

Although the first emergence of pastoral economies in Mongolia remains poorly understood, recent decades of archaeological and ethnographic research point to a crucial role for mountain zones in this complex transition. The Altai Mountains form the western boundary of Mongolia and the eastern Eurasian steppes, stretching from Siberia in the northwest along the western edge of Gobi Desert in a southeasterly direction (Fig. 1). Their high peaks, ranging up to ~ 4500 m, trap precipitation traveling eastwards across the continent, creating a large rain shadow that contributes to the especially harsh, cold winters and warm, dry summers across the Eastern Steppe. The Altai Mountains themselves, however, receive more precipitation than surrounding regions—while their summer snowmelt results in productive summer alpine pastures. Modern herders in the region raise large numbers of domestic animals. The area has historically supported large populations of wild game, including argali sheep (*Ovis ammon*)*,* Siberian ibex (*Capra sibirica*), and red deer (*Cervus elaphus*), although the area’s larger fauna are severely threatened by poaching, habitat fragmentation, and climate change^6^.

Occupied by hunter-gatherers since at least the end of the Pleistocene^7,8^, the Altai Mountains also yield the Eastern Steppe’s oldest direct evidence for domestic animal use—in the form of burials of the Afanasievo culture, apparent migrants from eastern Europe that brought with them sheep, goat, and cattle just prior to ca. 3000 BCE^9–11^. Following this initial dispersal of domestic livestock, the mountain margins of the Altai and Khangai were occupied by semi-mobile pastoral herders of the Afanasievo and later Chemurchek cultures, who raised domestic sheep, goat, and cattle across the Altai throughout the 3rd and early 2nd millennia BCE^10–14^. These early herders may have been constrained from using drier intermontane desert and steppe zones by their lack of horse transport^12^. However, by the late Bronze Age, the introduction of domestic horses, which appear widely in sites of the Deer Stone-Khirigsuur Culture (ca. 1250–700 BCE) across northern and western Mongolia, brought about widespread cultural and economic transformations^15^. Based on available evidence, the turn of the first millennium BCE saw the emergence of a highly mobile, multispecies pastoral economy characteristic of East Asian pastoralism, including ephemeral habitations and the use of domestic horses for meat and dairy^10,12,16^. Across the later mid-first millennium BCE, mortuary features show that western Mongolia continued to host pastoral horse cultures like the Pazyryk (ca. 400–200 BCE) and the Xiongnu (ca. 200 BCE-100 CE)^17^.

While some of these key events, such as initial dispersal of domestic livestock and the introduction of the horse, are apparent from research at monuments and funerary sites, almost no non-ritual faunal assemblages predating the late first millennium BCE have been identified in Mongolia. This absence of direct insights into the subsistence base of early herding societies prevents a more comprehensive assessment of the role played by wild taxa or environmental change in the transition to pastoralism. Those few faunal assemblages that have been recovered from this period^12,16^ suggest a strong dietary emphasis on sheep, goat, cattle, and horse among Bronze Age herders. However, widespread iconographic depictions of wild fauna—including elaborate deer images found on many deer stones^18^ imply that wild game still played a role in early eastern Eurasian pastoral culture.

Recent applications of multimethodological archaeological science approaches have shown promise in expanding the range of information that can be garnered from organic material from early prehistoric assemblages in Mongolia^10,12^. As a result, comprehensive analysis of artifacts recovered from ice patches and their unique preservation conditions provide a promising line of inquiry into understanding the prehistoric use of mountain zones and animal economies of the region’s earliest herders. We conducted remote sensing and intensive pedestrian and horseback survey of melting ice at five locations near the mountain of Tsengel Khairkhan in western Mongolia, analyzing organic artifacts recovered from these localities using radiocarbon dating, collagen fingerprinting, and Scanning Electron Microscopy to reconstruct patterns of subsistence, tool manufacture, and cultural activities and assess the role of alpine zones and exploitation of wild fauna in the emergence of pastoral societies in East Asia.

## Results

### Remote sensing

**Figure 2:**
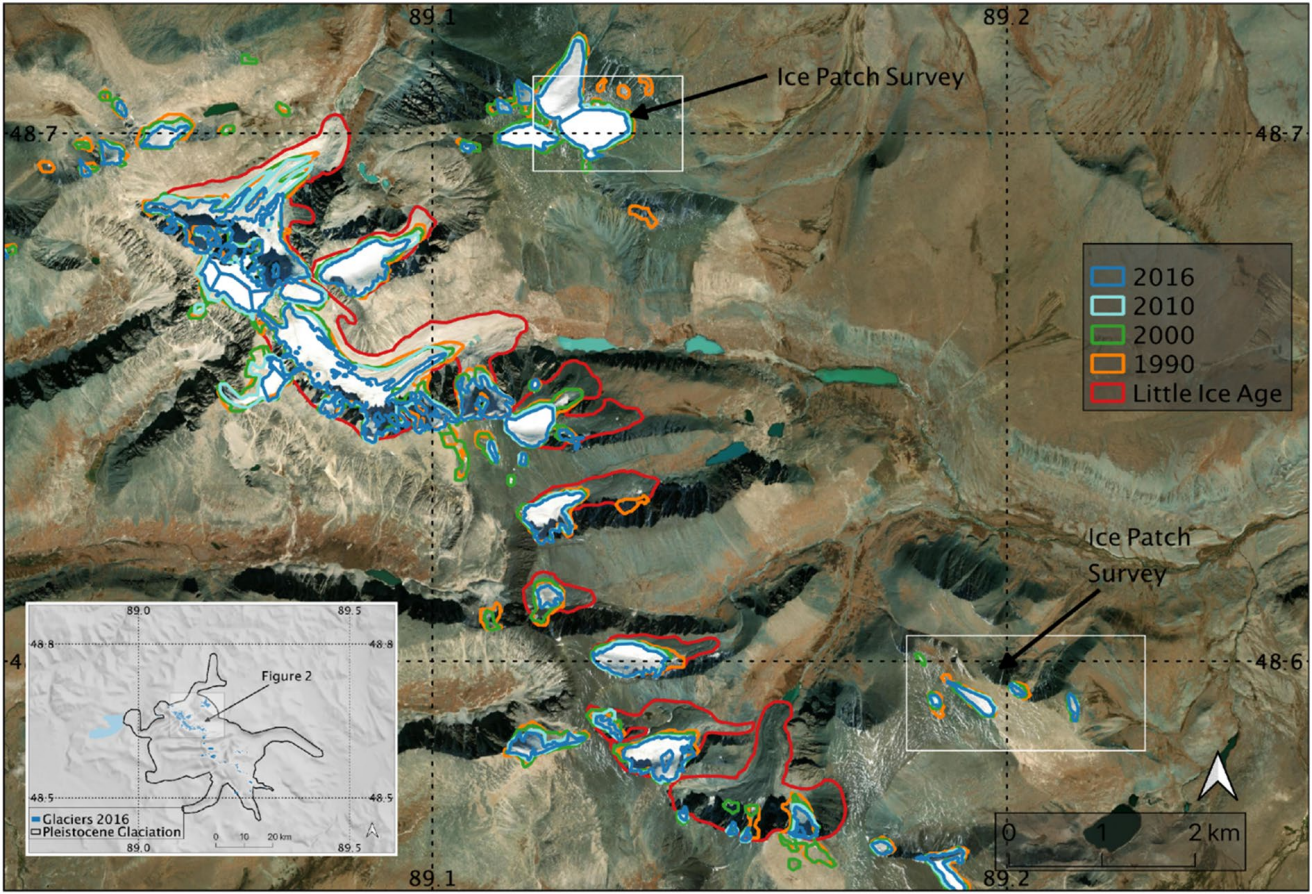
Ice extent in Tsengel region from 1990 through 2016, using glacier outlines Khairkhan from the Global Land Ice Measurements from Space (GLIMS) database^51,52^, modified from Walther et al.^53^ Ice Patch Survey boxes represent areas identified with more permanent ice (2010–2020) where we conducted intensive surveys of the maximum melt extent in August 2019. Inset shows the location of the study area relative to the boundary of the Pleistocene glaciation. Produced in QGIS 3.10 (http://www.qgis.org).

Analysis of remote sensing imagery identified several stable ice patches and glacial margins atop Tsengel Khairkhan and adjoining peaks. Comparing their present extent with imagery from the past three decades revealed a precipitous decline of more than 40% in total surface coverage of summer snow and ice extent at peak melt season since 1990 (Fig. 2, Table 1). The spatial patterning of the present coverage suggests that south-facing margins were most significantly impacted by the ice loss, especially at lower elevations.

**Table 1:** Estimated snow/ice extent in the study area, as measured through summer-season satellite imagery (LANDSAT 5 & 8 and Sentinel 2).

Year	Estimated area	% of 1990 total
1990	14.2 km^2^	–
2000	13.3 km^2^	93.7
2010	10.3 km^2^	72.5
2016	8.2 km^2^	57.7

### Pedestrian and horseback survey

**Figure 3:**
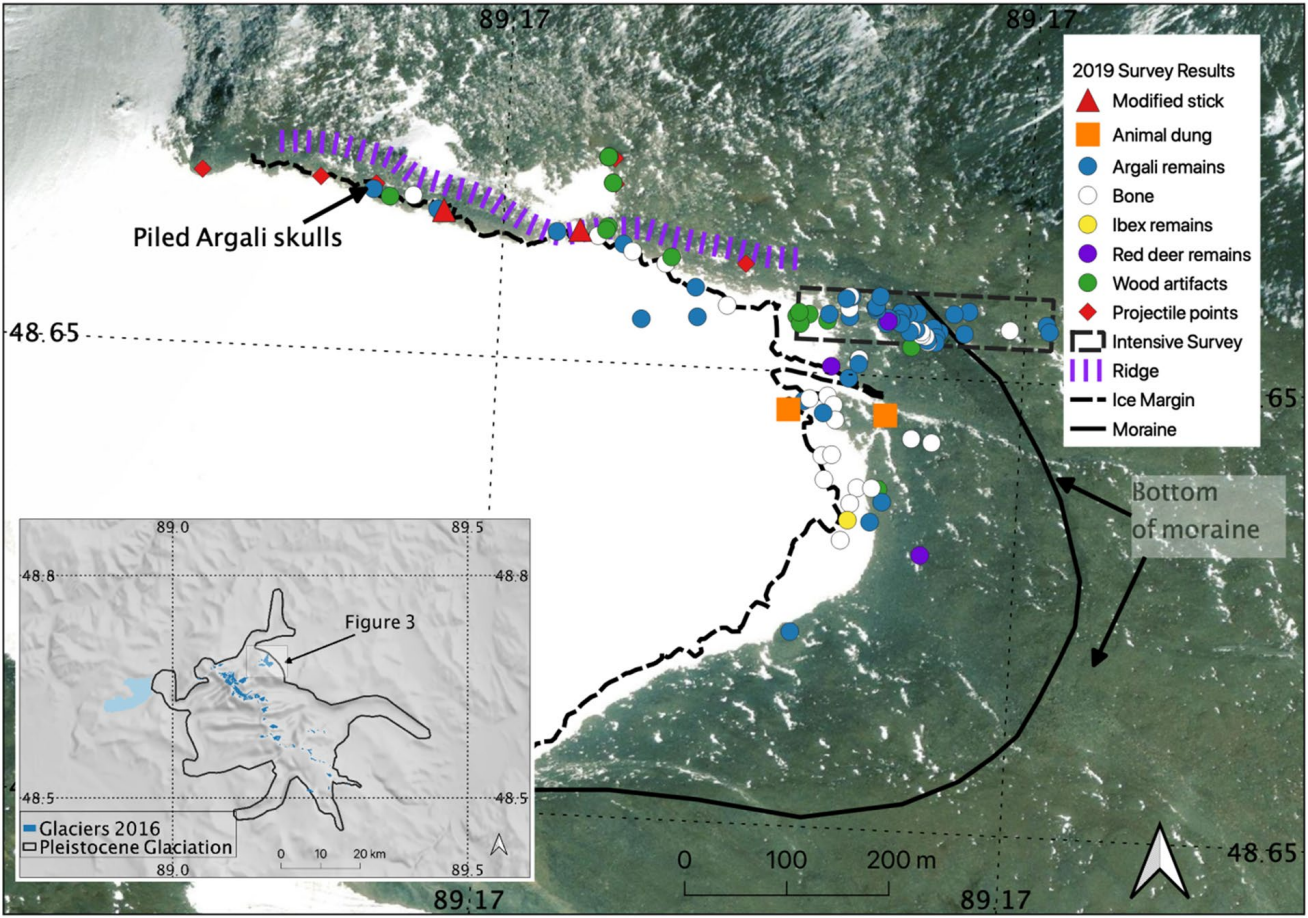
Results from pedestrian survey at Tsengel Khairkhan glacier. Imagery from ArcGIS High Resolution World Imagery dataset. Moraine represents maximum extent of Little Ice Age glaciation. Produced in QGIS 3.10 (http://www.qgis.org).

Our survey of five ice patches and one glacier margin revealed three localities bearing artifacts and ancient biological material, as well as one locality where faunal remains, with no direct evidence of a cultural origin, were recovered from periglacial pools frozen at the time of recovery (Supplementary Fig. S1). The most archaeologically productive locality we identified (Fig. 3) consisted of a small glacial ridge along the northeastern slope of Tsengel Khairkhan peak (which reaches an elevation of nearly 4,000 m). Here, local residents reported discoveries of willow arrow shafts, an antler projectile point, and metal arrow tips during a previous excursion (ca. 2010). Returning to the location of these discoveries—a small, V-shaped runnel formed by intersection of the glacier with a topographic prominence or subpeak that forms the glacier’s northern margin—we observed dozens of animal bones recently exhumed or actively emerging from the ice (Fig. 3). The bulk of animal bones observed were those of argali sheep (*Ovis ammon*), and were typically represented only by the horns or the cranium. Given the site’s large size and our difficulty in reaching the site, which spans an elevation range of 3500–4000 m, we were unable to comprehensively assess the total number of animal bones at the site during 2019 fieldwork. Within the glacier, large horn curls can be seen protruding from the ice many tens of meters from the current melt margin, suggesting that the total number of individuals may extend into the hundreds.

**Figure 4:**
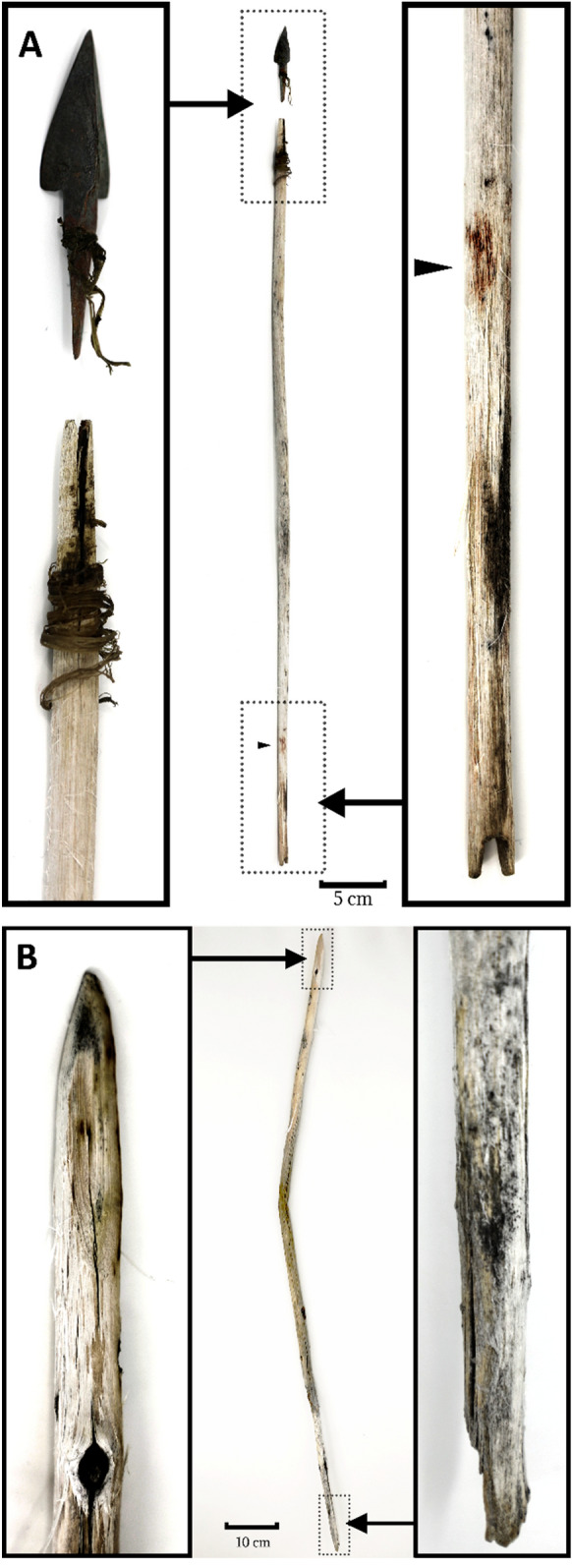
(**A**, top): intact, bronze-tipped and sinew-hafted arrow recovered from Tsengel Khairkhan (Artifact 35), dating to the late Bronze Age. The nock end has been painted with red pigment. Scale = 20 cm. (**B**, bottom): large dart or spear fragment (Artifact 27), narrowed at one end and with scarf joint at the other. Scale = 20 cm. Image: Peter Bittner and Mark Williams.

Focusing our documentation efforts on cultural materials actively melting from the ice margin, we identified 29 wooden artifacts at Tsengel Khairkhan, including 18 arrow shafts or shaft fragments, two spears/darts or dart fragments, three large anthropogenically modified sticks, and five otherwise unmodified segments of wood that may have been transported through human activity. These artifacts included one completely intact arrow tipped with a bronze, three-tanged arrowhead and hafted using animal sinew (Fig. 4), and a second willow arrow shaft tipped with an antler arrowhead that had broken into several fragments prior to recovery. We also identified one bone arrowhead, two large fragments of animal sinew, and an isolated find of a badly weathered, four-tanged iron arrowhead (Supplementary Fig. S2).

To assess the impact of ice melting on artifact preservation and taphonomic processes at the site, we conducted an intensive survey of a ~ 20 m x 250 m area by walking transects spaced 5 m apart, extending from just below the actively melting ice margin downslope until no more faunal or ancient biologic material was found. Within this zone, we recorded every visible faunal specimen in situ*,* assigning it a weathering score based on Behrensmeyer^19^. Results of this survey indicate that material upslope has been only recently exposed—with most objects in this area exhibiting a score of 2 or less. In contrast, most objects more than 100 m from the 2019 ice margin were badly weathered, with a score of 3 or 4 being most common. Radiocarbon dates of argali bone and horn from within this transect suggest that the Tsengel Khairkhan assemblage is a palimpsest of accumulated material spanning a long range of time, with upslope ice deposits dating at least as far back as the early Bronze Age (ca. 3840 ± 23, OxA-39960, ca. 2342–2202 cal. BCE at 95.4% probability), but some specimens dating as young as the late historic or modern era (ca. 131 ± 20, OxA-39635, ca. 1680–1940 cal. CE, 95.4% probability). The furthest dated downslope specimen was dated to the fifteenth-sixteenth centuries CE, and was found near the LIA moraine (Supplementary Fig. S3). While downslope specimens exhibit a greater degree of weathering, wide variability in this overall pattern may suggest that localized topographical variations in the scree slope (such the micro-environments described by Pilø et al.^4^) exert a significant influence on taphonomic preservation –with some older objects preserved in low-lying, snow-covered microregions exhibiting better preservation than younger objects. Because of their location downslope, many artifacts may have also been exposed in prehistory, and secondarily transported downslope from an initial location higher on the glacier. Thus, at Tsengel Khairkhan, accumulated materials spanning nearly three millennia are emerging from the ice margin through recent melt activity. The rapid degradation of perishable artifacts and faunal remains after emergence from the ice reinforces the urgency of this kind of archaeological reconnaissance.

### Radiocarbon dating and cultural patterns

We directly radiocarbon dated 34 archaeological and paleobiological specimens recovered from Tsengel Khairkhan Glacier (consisting of 14 wooden arrow shaft fragments and 2 wooden dart/spear shaft fragments, 3 large worked sticks, 5 pieces of wood, one piece of sinew lashing from a hafted arrow, one piece of unmodified sinew, 5 argali horn/bone specimens, and 3 wooden and bone artifacts previously recovered from the glacier by area resident Mr. Bugibay Bekbolat, Tables S1 and S2). We also dated an animal-hair rope from Khultsuut Ice Patch 3 and a sheep/goat tooth from Khultsuut Ice Patch 1 (Supplementary Fig. S5). Our radiocarbon dates demonstrate that the ice at these associated localities contains a near-continuous cultural and paleobiological record for the region, stretching from the 3rd millennium BCE through the present day (Supplementary Fig. S6). At Tsengel Khairkhan, the ice melt along the northeast margin of the glacier has exhumed artifacts dating to the early first millennium BCE and beyond. Those artifacts along the northernmost margin, which have been trapped by the small subpeak ridge, are younger, between ca. 0–1000 CE (Supplementary Fig. S4). Artifacts and faunal specimens recovered from the eastern margin of the glacier, particularly in the small snow-filled terraces and undulations, generally dated to more recent periods, ca. 1500 CE-present.

### Species identification of plant and animal taxa

Our taxonomic identification of bone, antler, and soft tissue from culturally-modified artifacts at Tsengel Khairkhan indicates that deer and sheep raw materials were selected for projectile production. While it is perhaps an unsurprising find that antler arrowheads were made from deer antler (Cervidae sp., probably red deer), the bone arrowhead appears to have been made from the bone of *Ovis* sp*.* (either domestic or wild argali sheep, Table S3). Collagen fingerprinting also shows that the sinew chosen for hafting the bronze arrow was made from deer tissue, a choice that seems notable given the likely availability of domestic animal taxa (such as sheep, cattle, and goat). Of the unmodified sinew scraps recovered from the melting ice, one proved to be non-mammalian, perhaps from a large bird, while another comes from *Ovis* sp. While there is no definitive evidence indicating that this sinew material is cultural in origin, it could have been discarded during butchery of argali sheep near the ice margin. The argali sinew is among the oldest biological material identified at Tsengel Khairkhan (3275 ± 28 ^14^C yr BP, OxA-39827, ca. 1616–1461 cal. BCE at 95.4% probability). Finally, our analysis of an animal-hair rope artifact from Ice Patch 3, dated to the early Middle Ages (1582 ± 18 ^14^C yr BP, OxA-39828, ca. 429–545 cal. CE), using Scanning Electron Microscopy (Supplementary Appendix A) suggests that the fiber artifact is likely made from hair of the Bactrian camel (*Camelus bactrianus*).

Our analysis of wooden artifacts through comparison with known materials from area wood taxa indicates that nearly all specimens identified during 2019 fieldwork were made of willow (*Salix* sp., Table S1). This observation includes both arrow shafts and larger spear or dart shafts dating from the late Bronze Age (ca. 1200 BCE), through the first century CE. The only exception to this pattern comes from larger sticks with worked ends (possible “scare sticks” used to stick upright in the ice and guide animal movement during hunting), which were sometimes made from other locally-available plant taxa such as buckthorn (*Hippophae* sp.) and elm (*Ulmus* sp.).

## Discussion

### Weapon manufacture

Projectiles recovered from Tsengel Khairkhan provide important new insights into the process of projectile and weapons manufacture for hunting in early pastoral societies. Nearly all of the recovered wooden projectiles or projectile fragments, spanning the Bronze to the late Iron Ages, were made from willow (Table S1). Willow is a light and strong material, with several species native to northern Eurasia and widely available in the Mongolian Altai in the lower alpine belt^20^, and it was commonly chosen for both darts and arrows in ice patch archaeological assemblages across the globe^21,22^.

The arrow from Tsengel Khairkhan (Artifact 35, Fig. 4A) is the first nearly-intact Bronze Age projectile that has been recovered from the eastern Eurasian steppe. The tripartite bronze arrowhead is hafted into a thin willow branch using a split shaft, evenly worked to roughly 8 mm in diameter. Interestingly, despite the easy availability of similar raw materials from domestic species in late Bronze Age pastoral societies, the bronze head has been carefully hafted to the shaft using deer sinew—a choice that may reflect cultural preference rather than practical concerns. Radiocarbon dating of two material types from this specimen, the sinew and the willow, produced a slightly older radiocarbon date (2736 ± 18 ^14^C yr BP, OxA-39842, ca. 916–831 cal. BCE) for the willow compared to the sinew (2625 ± 19 ^14^C yr BP, OxA-39843, ca. 821–791 cal. BCE), which indicate that the arrow shaft was made of older wood, or curated/reused after its initial creation. This technology is consistent with the region’s sparse archaeological record for the early first millennium: arrowheads from the late Bronze Age Karasuk culture, in southern Siberia (ca. 1400–1000 BCE), tend to be made primarily of chipped stone or bone^23^, but similar tanged bronze arrowheads have been reported from the seventh century BCE burials at Arzhan 2^24^. Recent excavations of a cenotaph from the site of Jargalantyn Am in north-central Mongolia revealed nearly identical tripartite bronze arrowheads with remnant hafting^25^ (Supplementary Fig. S7), and our radiocarbon date of horse remains from this burial yielded a slightly younger date of 2562 ± 21 ^14^C yr BP (OxA-39944, ca 803–592 cal. BCE). The Tsengel Khairkhan arrow shaft itself shows that the area of the fletching was decorated with red pigment, a practice seen on late first millennium BCE arrows from the Russian Altai, that may be a means of indicating ownership or demarcating arrow type^26,27^. No fletching was recovered from the arrow, which may have been stripped off or lost post-depositionally. A small divot in the arrow’s tip may reflect impact damage.

Our discoveries reveal that additional hunting technologies—the javelin or spear—were used in the Altai long after the initial adoption of the bow, perhaps in ambush/drive hunting. Near the top of the small subpeak forming the glacier’s northern margin, we discovered one intact spear or dart (Fig. 4B), and a second fragment with a smooth, even shape and a wide diameter of roughly 18 mm. The spear/javelin or dart– which was found wedged between two large rocks—has been narrowed at one end, while the other terminates at a beveled scarf joint, a traditional means of attaching willow shafts in Mongolia^28^, which may have originally joined to a foreshaft. Radiocarbon dating fixes these two specimens in the late Iron Age, between 20 cal. BCE—80 cal. CE, the later decades of the Xiongnu Empire (ca. 200 BCE-100 CE, Table S1). These objects may have been used in ambush hunts, where game were driven up the glacier’s edge and ambushed by hunters lying in wait among the tall rocks.

An important element of the Tsengel Khairkhan artifact assemblage is the relatively high frequency of bone and antler arrowheads (which including three found during survey, and one previously identified specimen). These finds outnumber metal projectiles at the site by a ratio of 2:1, showing strong technological continuity across thousands of years in the eastern Altai. Radiocarbon dating of three of these specimens demonstrates their use from at least the early first millennium BCE (2716 ± 24 ^14^C yr BP, OxA-40153, ca. 907–811 cal. BCE) through the early second millennium CE (938 ± 17 ^14^C yr BP, OxA- 39,845, ca. 1039–1158 cal. CE). Two antler arrowheads appear to have been hollowed out and “friction-fit” onto the end of a wooden shaft (Supplementary Fig. S8). A third bone arrowhead fragment, dating to the Xiongnu Era (2024 ± 32 ^14^C yr BP, AA-114930, ca. 106 cal. BCE-108 cal. CE), does not retain its lower portion, and so the system of attachment or hafting could not be ascertained. Ease of manufacture and greater availability of antlers may have encouraged the use of organic points over metal counterparts for hunting.

### Hunting strategies and butchery

The spatial concentration of projectiles near the ridge that forms the glacier’s northern margin, along with the presence of other modified wooden artifacts in this area, could be a reflection of downslope transport—but it appears likely that ancient hunters utilized this natural landform to drive and ambush big game, primarily argali sheep. Almost all of the projectiles recovered by our project originated from the runnel formed by the intersection of the ice margin with the ridge’s southern slope (Fig. 4). Additionally, along this ridge we recovered three anthropogenically modified sticks, which appear to have been sharpened, perhaps to facilitate their planting upright in the snow as is sometimes used in alpine game drives elsewhere in Eurasia^4^. So-called “scare sticks” are typically placed in linear arrangements to block escape routes and control the movements of prey animals, who will instinctively avoid them as they flee pursuers. At Tsengel Khairkhan, we recovered these sticks only along the upper edge of the ridge, where we hypothesize they were driven into the ice or rocks to funnel sheep into the channel formed by the glacier margin and the ridge, to be ambushed by hunters hiding in the rocks above. Some of the modified sticks we recovered from Tsengel Khairkhan are among the oldest artifacts at the site, dating to ca. 1425–1296 cal BCE (3098 ± 21 ^14^C yr BP, OxA-39837, 95.4% probability) and ca. 1050–927 cal. BCE (2839 ± 18 ^14^C yr BP, OxA-39846, 95.4% probability), respectively. Other evidence for hunting, including nearly-intact arrows, comes from a separate ice patch along a small north-east facing terrace, where good cover may have given hunters more success with a stalking strategy. Here, hunters appear to have been originally positioned within bowshot of the patch, where arrows were apparently lost in the ice after being released.

Surface survey strongly suggests that the primary animal taxon hunted at Tsengel Khairkhan was the argali sheep, comprising the overwhelming majority of identifiable visible finds; however, we also identified the remains of red deer (*Cervus elaphus*) and Siberian ibex (*Capra sibirica*). At present, all three taxa inhabit high elevation zones in the study area^29–31^. Modern argali occupy a variety of ecozones, including steppe, semi-desert, desert, and mountain environments, across elevations ranging from 300 m to 5750 m^30^. Modern argali distribution is also shaped by competition from livestock and other wild ungulates, predation, and availability of critical resources such as water and forage^30,32–36^. The high frequency and visibility of argali remains at Tsengel Khairkhan imply that argali were relatively abundant at the study site in antiquity, and more detailed future study may help understand the relationship between prehistoric argali populations and climate dynamics at high altitudes in the Altai.

While we did not conduct a comprehensive zooarchaeological study, the predominance of cranial elements and horns visible at the surface suggest that decapitation at the kill site was a standard practice at Tsengel Khairkhan from the first millennium BCE. Interestingly, cranial or mandibular elements also comprised the vast majority of identified materials at ice patches in the Greater Yellowstone area of the United States^5^ where bighorn sheep (*Ovis canadensis*) skulls and horn cores are the predominate faunal element at ice patch sites. Future zooarchaeological analysis will be necessary to assess evidence for butchery on argali remains at Tsengel Khairkhan, but it is possible the presence of large and unwieldly horns and antlers and the difficulty of mountain transit factored into the choice of leaving behind cranial elements. Our isolated finds of sinew may support the idea that animals were at least partially butchered near the glacial margin after a successful hunt.

### Ritual activity

**Figure 5:**
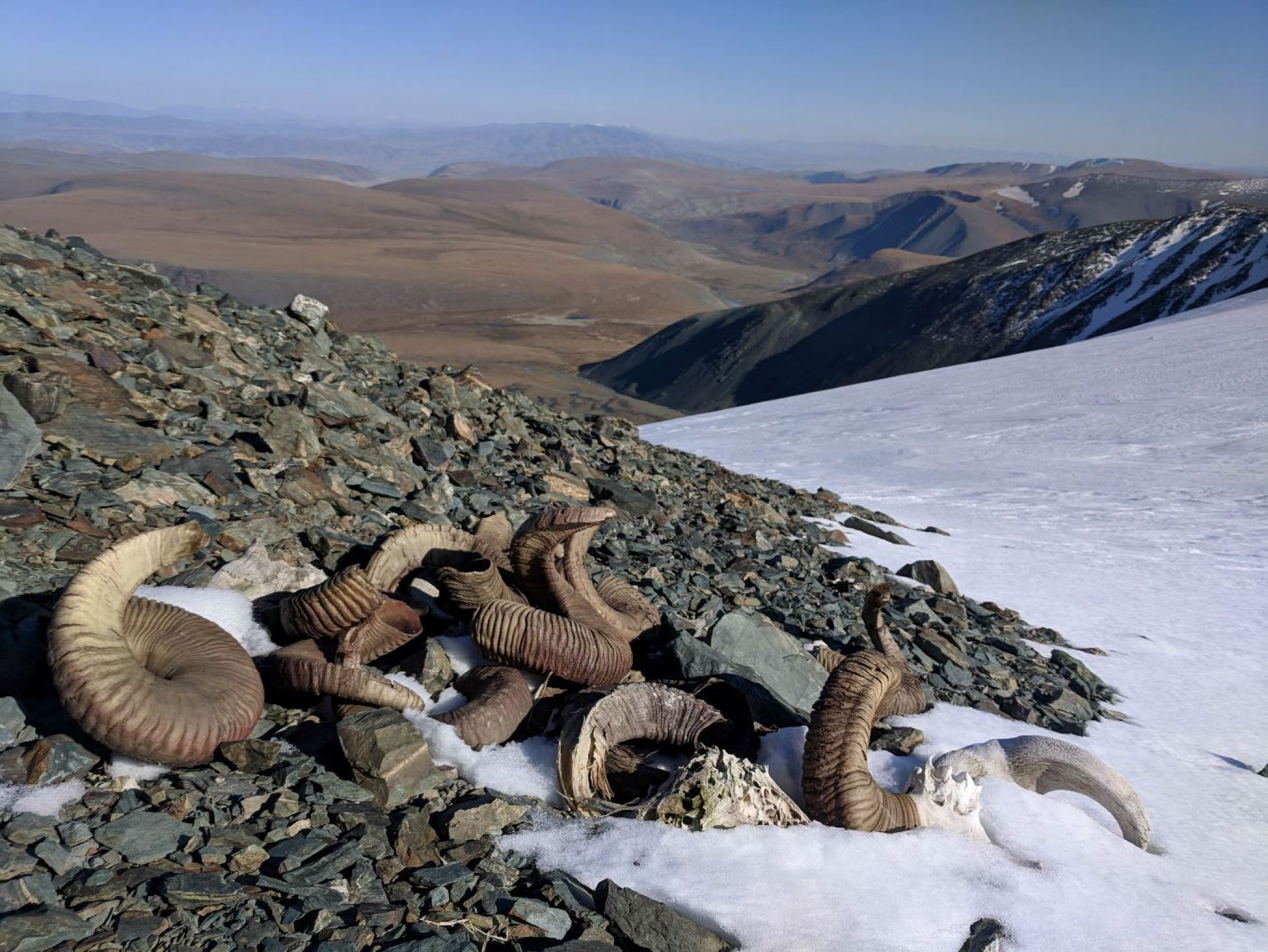
Piled argali horn sheaths and horn-cores, melting out of the ice margin at Tsengel Khairkhan. Image: William Taylor.

Our results also reveal that hunting activities played an important role in the worldview and spiritual beliefs of early pastoralists, as revealed by the treatment of argali sheep skulls at Tsengel Khairkhan (Fig. 5). Nearly all of the argali remains we encountered on the mountaintop were either intact skulls, fragmented skulls, or horn sheath curls. Recognizing the practical considerations in leaving behind the cranium, the occurrence of individual piles of skulls melting directly out of the ice nonetheless provides clear evidence of their intentional placement. In contemporary Mongolia, the head of an animal is imbued with special significance, and the skulls of a deceased horse are often placed at a high mountaintop *ovoo,* or stone cairn^37^. During the late Bronze Age, the ritual placement of the head, neck, and hoof bones of deceased horses was standard practices at burials and monument sites of the DSK complex (ca. 1200–700 BCE)^38,39^. We radiocarbon dated one argali skull pile at Tsengel Khairkhan, consisting of the horns and crania of at least five individuals, to ca. 250 CE (1816 ± 20 ^14^C yr BP, OxA-39958, 169–325 cal. CE). Based on their size and the pattern of horn sheath growth rings, all of the skulls in this arrangement seem to be large, older males, which might have been preferentially hunted for their larger body size and/or cultural significance. While we cannot rule out the possibility that the piles we identified at Tsengel Khairkhan were created by later visitors using ancient skulls, our findings raise the possibility that following a successful hunt, early pastoralists afforded similar respect to their prey, leaving the skull of the animal atop the mountain intentionally out of respect or spiritual practice, as observed in ice patches at the Greater Yellowstone Area, USA, with bighorn sheep^5^.

### Other cultural activities

Archaeological finds from lower-elevation patches at the nearby location of Khultsuut also show the use of mountain zones for other activities, including transport and herding. Present-day herders use the area around Ice Patches 1 and 3 for pasturing sheep and goats during the summer months. It is likely that these high mountain patches—fed by melting snow and ice—provided a seasonally-productive summer pasture for pastoral purposes in the past as well. Radiocarbon dating of a single sheep tooth from Ice Patch 1 (identified as *Ovis* through collagen fingerprinting) indicates that this specimen is of modern origin but at least several decades old (1.21608 ± 0.00308 F^14^C, or ca. 1959–1985 using CaliBomb, NH Zone 1). Other materials from near this patch, including a horseshoe and nails, appear to indicate horse-riding activity in recent centuries. At the slightly higher elevation Ice Patch 3 (~ 3250 m), we also identified a rope fragment made of animal hair, identified as Bactrian camel through SEM, from the fifth/sixth centuries CE. This artifact could represent a component of a bridle/halter, as similar artifacts have been recovered from Xiongnu tombs^40^, or could have been used for securing pack bags during transport or hunting.

### Role of mountain zones in emerging pastoral societies

By providing some of the first well-preserved organic materials from Mongolia’s late Bronze Age pastoral societies in the Altai, our discoveries reveal a significant and previously undocumented role for big game hunting in the lifeways and belief systems of the eastern steppe’s early herders. Our results demonstrate that use of high-mountain zones has played a crucial role in pastoral adaptations since at least ca. 1200 BCE in the Altai Mountains.

This research presents the first direct evidence for Bronze Age organic weapon manufacture and hunting strategies in the Mongolian Steppe. Only stone and metal arrowheads are typically recovered from surface surveys, but our glacier finds show that antler and bone arrowheads—made from deer antler and bone of *Ovis* sp.—were commonly used, perhaps even preferred, for hunting since the early first millennium BCE. Despite the availability of raw material from domestic animals, the skeletal material and soft tissue of wild animals was selected for projectile point production (in tandem with willow, which appears to have been the favored material for both arrow and spear/dart shafts across our sample period). Hunting of argali was also not a purely pragmatic act—piles of skulls and horn sheaths, formed intentionally along the glacial margin, appear to represent intentional post-hunt offerings or spiritual behavior. Together, this work shows that big game hunting was an integral aspect of early herding life.

**Figure 6:**
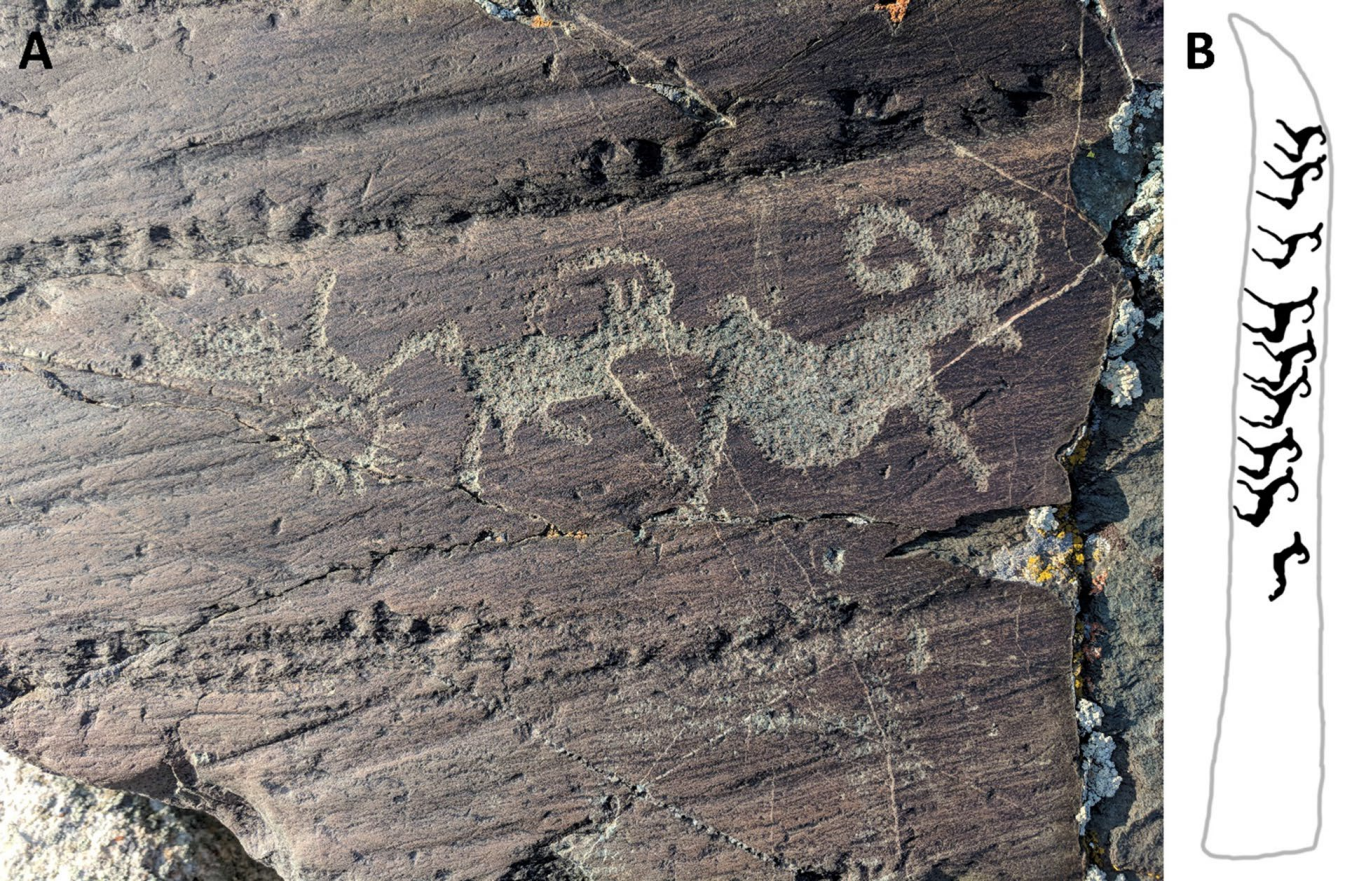
(**A**, left) A petroglyph panel at Khultsuut depicts argali sheep and ibex. (**B**, right): a side panel of what may be a herd of argali sheep adorns the side of deer stone from Arkhangai Province, Bayan-Tsagaan Gol (modified from Volkov^54^). Image: William Taylor.

Radiocarbon dating of projectiles recovered from the Tsengel Khairkhan glacier places many of these artifacts prior to ca. 800 BCE, linking them with the late Deer Stone-Khirigsuur culture^15,38^. Monumental standing stones of this culture are often decorated with large deer, and deer (probably *Cervus elaphus*) and wild game form an important component of Bronze and Iron Age assemblages in adjoining regions of Central and Northeast Asia^41,42^. To date, however, no archaeological projects have identified evidence for wild game consumption in DSK or other early Mongolian pastoral cultures. However, our inference of a major cultural and economic role for *Ovis ammon* across the hunting/herding transition in the Altai and other areas of eastern Eurasia finds some support in the petroglyph record, where animals such as argali and ibex are frequently depicted^43^ (Fig. 6). Argali and ibex images are commonly carved directly on late Bronze Age monuments known as deer stones^14^ and at least one deer stone displays a side panel entirely decorated with argali images (Fig. 6).

Our findings indicate that the frequency of these taxa in rock art likely reflect key economic roles not previously identified in the archaeofaunal record. Argali sheep are rarely distinguished from the smaller domestic *Ovis* in archaeofaunal assemblages, and as such, their economic role may have escaped the notice of scholars working with badly fragmented and rare zooarchaeological assemblages. For example, recent analysis of an early second millennium BCE occupation site near Tsengel Khairkhan dated to ca. 1650 BCE showed a predominance of *Ovis* remains, initially presumed to be domestic sheep^12^. In southeastern Kazakhstan, argali sheep remains occasionally occur in early pastoral assemblages spanning the first and second millennia BCE^41^. Future work, relying on careful morphological comparisons or DNA, may be necessary to assess the frequency of argali sheep in early pastoral dietary assemblages, which are often dominated by *Ovis* remains but typically presumed to be domestic sheep.

### Implications of climate change and poaching

As the climate warms, Mongolia and other subarctic zones are experiencing disproportionate impacts from resulting environmental change. Paleoclimate data from the Altai region are generally too sparse and complex to easily correlate with ancient cultural patterns at Tsengel Khairkhan on the basis of existing data (Supplementary Appendix B, Supplementary Fig. S10), but modern data make it clear that ice loss poses a severe threat to contemporary herding lifeways. In northern Mongolia, our preliminary study of ice patches in the Ulaan Taiga protected region of the Sayan Mountain range along the Russian-Mongolian border found domestic reindeer herders were struggling to cope with drying pastures and health impacts to reindeer from the loss of ice patches^28^. Similarly, the Altai region appears to be experiencing pronounced levels of summer melting contributing to retreating glacial ice^44^.

In the area near Tsengel Khairkhan, 2019 discussions with area herders suggested that recent melting had exposed ice to levels unseen in recent memory—causing them to worry about the future water levels and viability of area pastures. This interpretation is corroborated by our analysis of historical glacial boundaries. Peak melt season snow cover in the broader Tsengel Khairkhan region covered an area of roughly 14.2 square kilometers in 1990 declining to approximately 10.3 square kilometers in 2010, and 8.2 square kilometers in 2016—a decline of more than 40% in a period of two decades (Table 1). As a recent global analysis of glacier mass balance identified particularly low variation in glacial mass balance in Central Asia, it is likely that interannual variability in ice levels is primarily due to warming summer temperatures across the region^35^.

While these impacts of summer warming on Altai ice are pernicious, the present study also indicates that diminishing wildlife populations portend serious deleterious impacts on the future of pastoral herding in the Altai. Ice patch research in other areas of the world has shown that mountain hunting served as a bulwark against periods of low productivity or stress^45^, even during colder periods where mountain zones might be expected to be less productive for human use. Our discussions with area residents suggested that during the late twentieth century, big game animals were regularly seen atop Tsengel Khairkhan, but have not been observed there in decades. Big game populations have declined dramatically across Mongolia during the last thirty years, primarily due to illegal poaching driven by international wildlife markets^6,46^. The Altai argali sheep is currently classified as Endangered following International Union of the Conservation of Nature (IUCN) criteria, and populations are highly fragmented across the country^47^. The situation is exacerbated by poorly regulated ‘hunting tourism’ and an opaque permitting system^48,49^. In 2019, Donald Trump, Jr. was widely criticized for a trip to the study region in which two argali sheep were hunted and exported using a permit that was not issued until after the hunt was completed^50^. This intense hunting pressure, along with a warming climate and changes in livestock density have fragmented the distribution of argali in Mongolia, creating small, isolated populations that are more prone to extinction.

Our new findings demonstrate that the decline of wildlife populations is not only a conservation concern for species like argali, but also a threat to the viability of pastoral lifeways. Archaeological materials from Tsengel Khairkhan show that since the Bronze Age, herding societies have relied upon big-game hunting in mountain zones to provide additional subsistence resources, and that these species have also played a central role in ceremony, identity and belief systems. Argali hunting has therefore remained a key aspect of lifeways in the Altai for at least 3000 years. The modern loss of big game populations, and accompanying restrictions on legal hunting for residents, may compound issues related to climate change and ice loss and negatively impact the viability and resilience of pastoral lifeways. As populations decline further, the loss of big game will likely continue to reduce the region’s ecosystem functioning and viability for eco-tourism.

## Conclusion

While challenges in preservation have to this point prevented a clear assessment of the role of montane zones in the emergence of pastoral societies in eastern Eurasia, results from ice patch archaeological survey show that alpine hunting played an important role in pastoral subsistence strategies since at least the final Bronze Age. Our results provide the first direct insights into projectile construction and hunting strategies of the late second and early first millennium BCE, indicating that perishable antler and bone points were often used for hunting (along with sinew hafting and willow shafts). Projectile artifacts show that mountain glaciers and ice patches were used for ambush hunting with arrows, darts or spears, which were in use during the late Iron Age. The impact of hunting was not limited to subsistence—raw material selection choices and the placement of argali sheep skulls and horn sheaths suggest a post-hunt ritual treatment, underscoring the impact of big game hunting in cultural or spiritual beliefs. As the climate warms and big game populations continue to decline from overhunting and illegal wildlife trade, the loss of mountain ice and wildlife will likely undercut the resilience of traditional herding lifeways in a rapidly changing world.

## Methods

Using publicly available satellite (LANDSAT 7) imagery taken during the late summer (August and early September), we analyzed the area for ice patches that appeared to be permanent during the period 2010–2018 and exhibited a flat forefield potentially conducive to artifact preservation. From this dataset, we identified five ice patches of interest. Through discussions with local collaborators, we also identified a sixth locality along the margin of the Tsengel Khairkhan glacier itself, where climbers reported finding wood and antler projectiles during prior summers. For pedestrian survey, we surveyed the margin of maximum melt extent (at the peak of the local melt season, in late August 2019) using a handheld GPS. At all ice patch locations, we conducted systematic surveys downslope from the ice margin until reaching the edge of lichen-free areas (which provide a proxy for regularly ice-covered zones) at 10 m transect intervals. At the Tsengel Khairkhan glacier, due to logistical constraints we were only able to conduct two 10 m transects downslope from the ice margin, and one area of full coverage survey using 5 m transects (Fig. 4). In the field, each artifact or ecofact was photographed in place using a digital camera and documented with GPS at a precision of ± 3 m, before being collected, dried, and stabilized for transport using acid-free conservation packaging. Unless such samples were collected for radiocarbon dating (n = 5) or collagen fingerprinting (n = 2), all non-artifact faunal specimens were left in the field as discovered. All artifacts are curated at the National Museum of Mongolia in Ulaanbaatar, Mongolia.

Next, we conducted osteological and biomolecular analyses of recovered artifacts to assess their age, preservation, and material composition. For bone objects, we made taxonomic identifications using reference images, and assessed the state of weathering for each recorded specimen based on criteria from Behrensmeyer^19^. All wooden artifacts and a selection of animal bones were sampled for radiocarbon dating at the AMS Laboratory at the University of Oxford, United Kingdom and the University of Arizona AMS Laboratory in Tucson, Arizona. Each radiocarbon date was calibrated using OxCal (https://c14.arch.ox.ac.uk/oxcal/OxCal.html) using the INTCAL20 calibration curve, and duplicated dates were combined in OxCal using the ‘R_Combine’ function. We sampled each artifact made of bone, antler, or sinew for taxonomic identification through collagen fingerprinting at the ZooMS Laboratory at the Max Planck Institute for the Science of Human History in Jena, Germany, following protocols outlined in Taylor et al.^12^.

Finally, we analyzed the impact of contemporary ice loss on the study area using remote sensing. To calculate ice loss in the region over the last three decades, we used glacier outlines from Tsengel Khairkhan for 1990, 2000, 2010, and 2016 available from the Global Land Ice Measurements from Space (GLIMS) database^51,52^. Glacier outlines were derived from Landsat 5 Thematic Mapper (TM), Landat 8—OLI, and Sentinel-2A imagery classified using a Near Infrared/Shortwave Infrared band ratio. For these glacier outlines, all glaciers identified in the band ratio needed to meet a minimum size threshold of 0.01 km^2^. Remote sensing analysis and figures were performed using QGIS 3.10 (http://www.qgis.org).

## Supplementary Information

Supplementary Information.
