# Supplementary material for: High altitude hunting, climate change, and pastoral resilience in eastern Eurasia
Source: Sci Rep. 2021 Jul 12;11:14287. doi: 10.1038/s41598-021-93765-w (PMC8275782; doi:10.1038/s41598-021-93765-w)
Supplement: Supplementary file 1 — Supplementary Information. [file 41598_2021_93765_MOESM1_ESM.docx]

**Supplementary Materials**

*
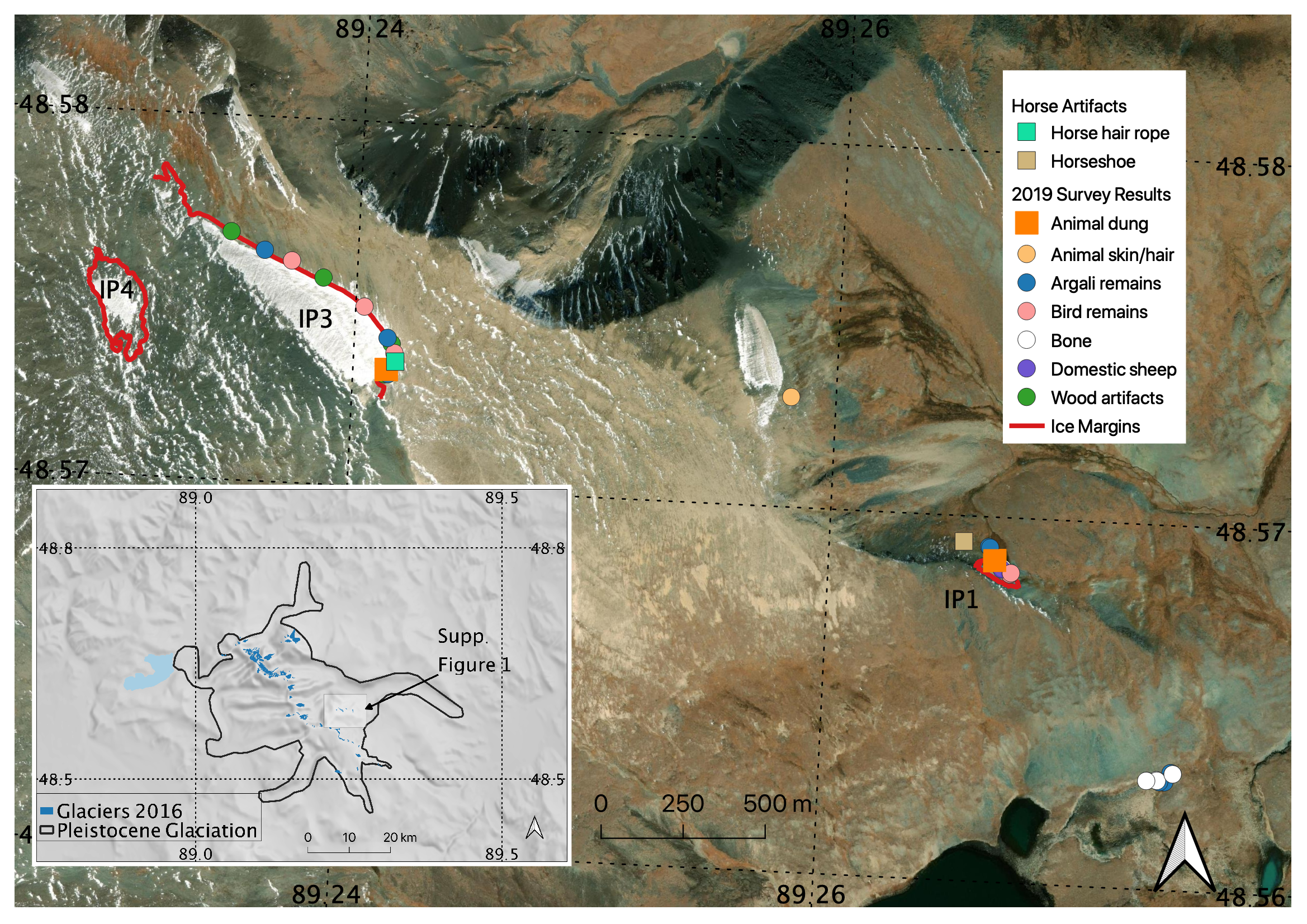
*Supplementary Figure 1. Results from pedestrian/horseback archaeological survey at artifact-bearing ice patches. Produced in QGIS 3.10 (http://www.qgis.org).

*Ice patch 1*

The first patch we surveyed, which is the lowest in elevation at 2940 m, yielded artifacts associated primarily with contemporary or recent pastoral herding. At the toe of the melted ice patch margin, we recovered two teeth belonging to domestic sheep/goat. The teeth, which were recovered together, consist of one upper and one lower fourth premolar. To the northwest of the patch margin, we identified an iron horseshoe and nails, which appeared to be modern in style (19^th^/20^th^ century).

*Ice patch 3*

While no obvious cultural or paleobiological materials were recovered at Ice Patch 2, directly to the northwest of Ice Patch 1, we identified Ice Patch 3, which yielded a wide range of biological material, including dung, feathers, bird bone, argali skulls, and argali horn fragments (Supplementary Figure 1). We also recovered a single piece of apparently unworked wood and a large segment of animal-hair rope (Supplementary Figure 5, Supplementary Appendix A).

**
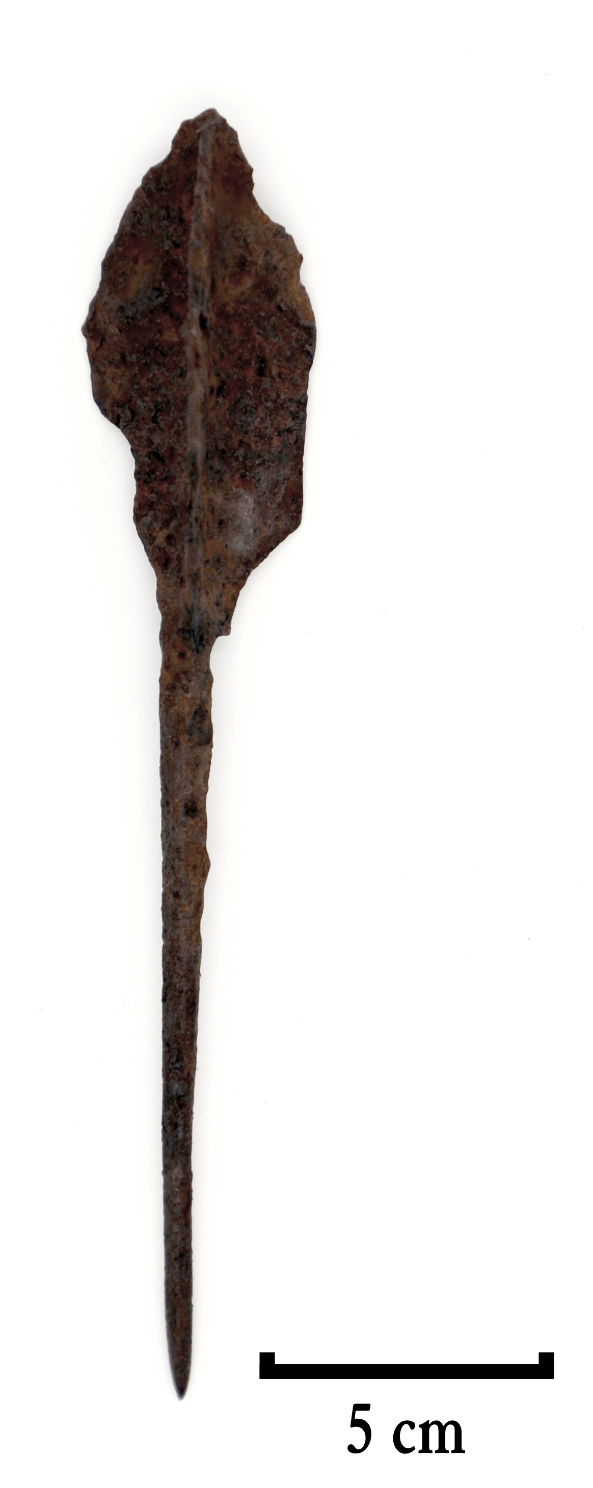
**

Supplementary Figure 2. Iron four-tanged arrowhead from Tsengel Khairkhan, likely dating to the first millennium CE. Image: Peter Bittner.

*
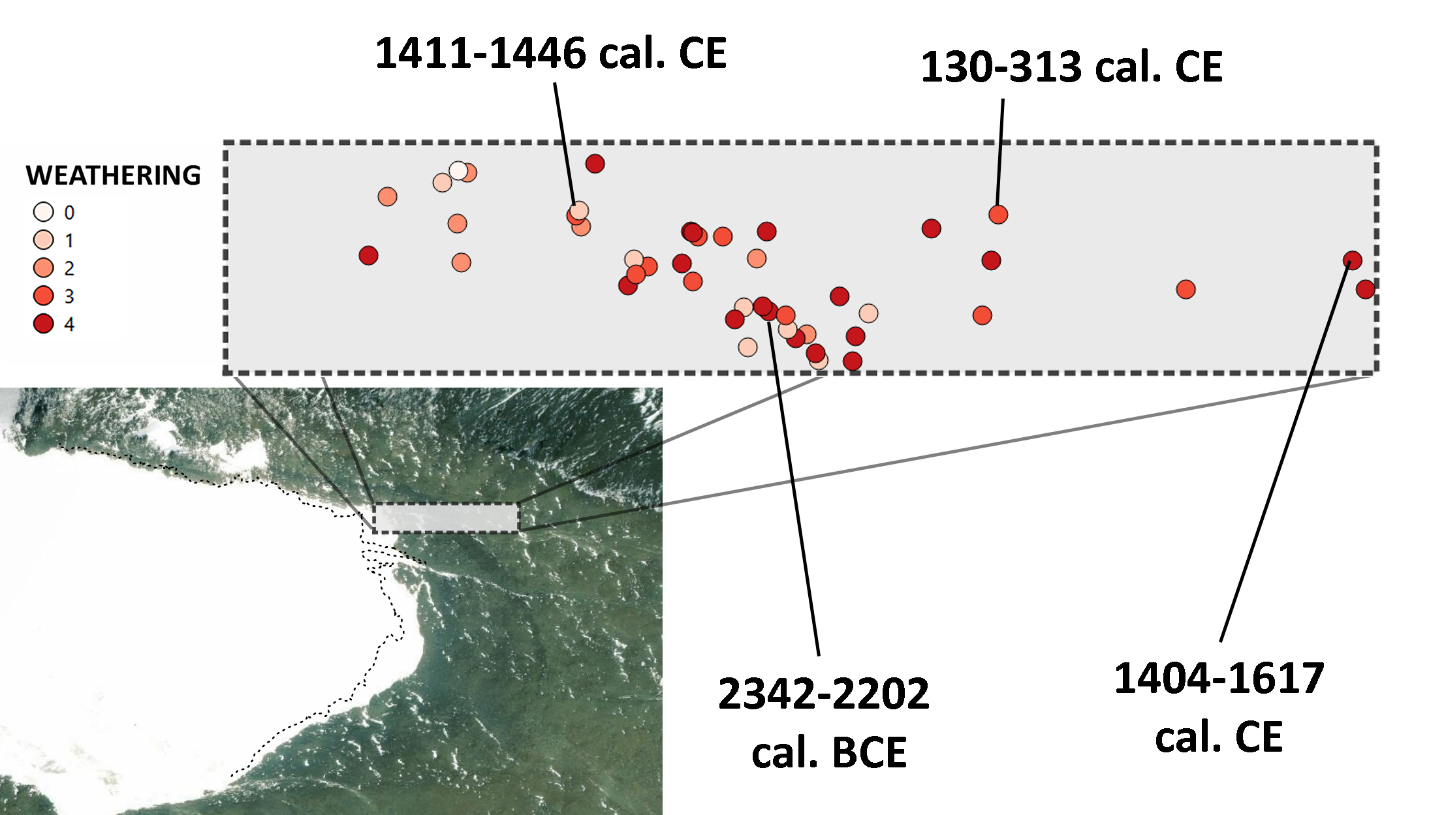
*Supplementary Figure 3. Results of full-coverage survey transect, showing weathering scores on faunal material following the categorical scoring system of Behrensmeyer (1978) along with accompanying radiocarbon dates (uncalibrated). Produced in QGIS 3.10 (http://www.qgis.org).


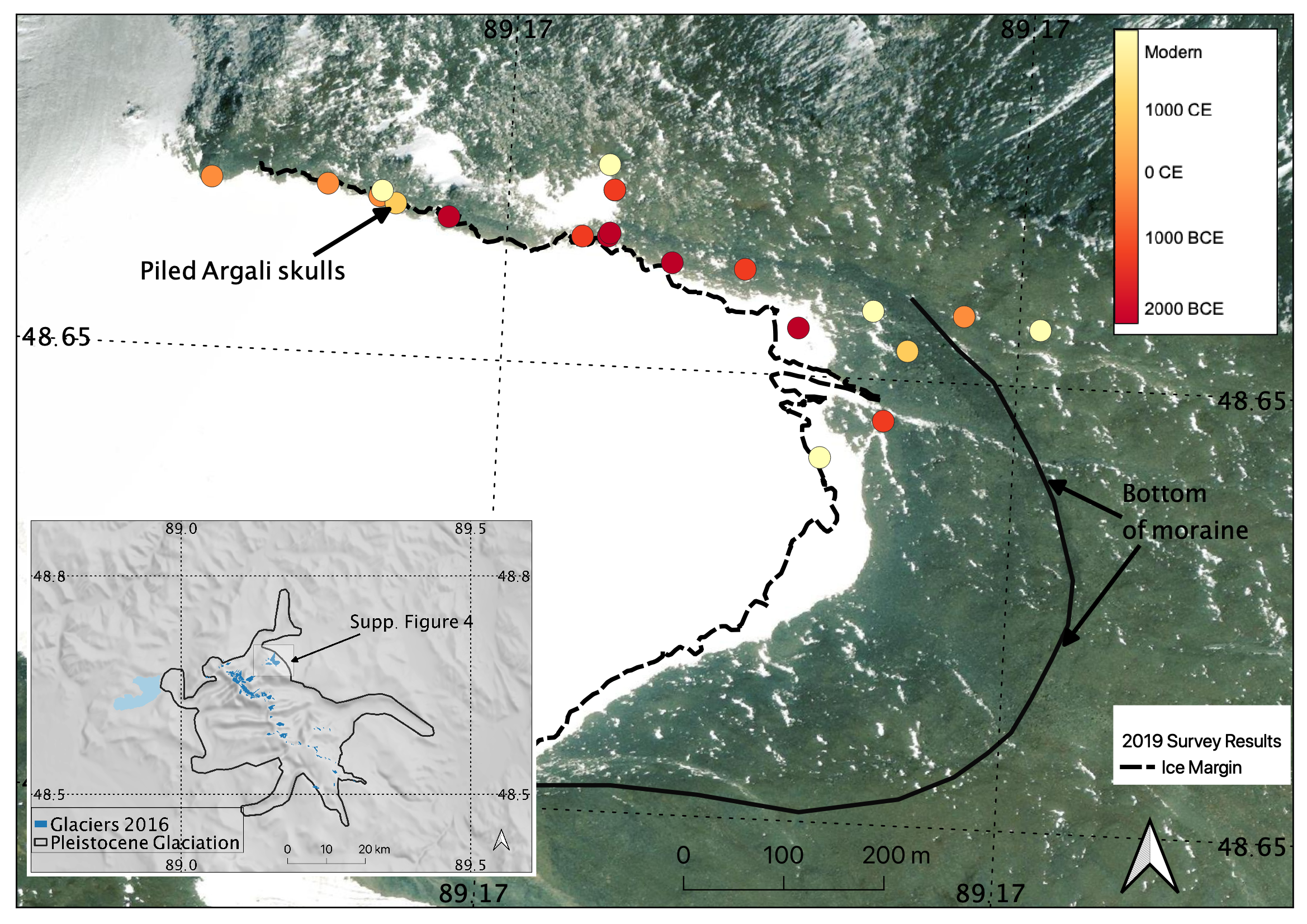


Supplementary Figure 4. Radiocarbon-dated artifacts at Tsengel Khairkhan, based on dates calibrated using INTCAL20, as compared to 2019 surveyed ice margin. Dates denoted with a (-) represent median calibrated years BCE, those without represent years CE. Produced in QGIS 3.10 (http://www.qgis.org).

**
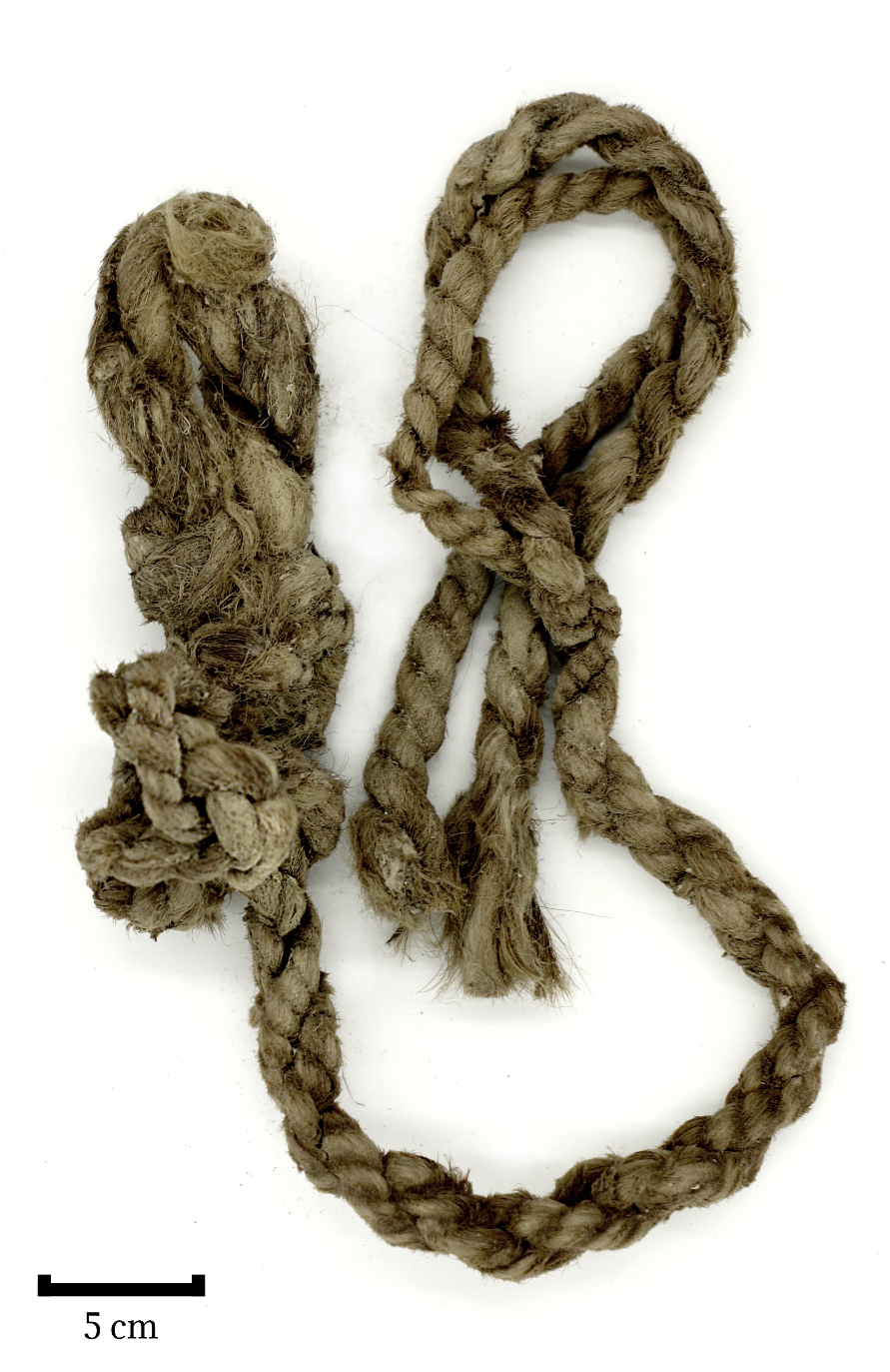
**

Supplementary Figure 5. Camel or horse hair rope fragment recovered from Khultsuut, Ice Patch 3, dating to ca. 500 cal. CE. Image: William Taylor.

*
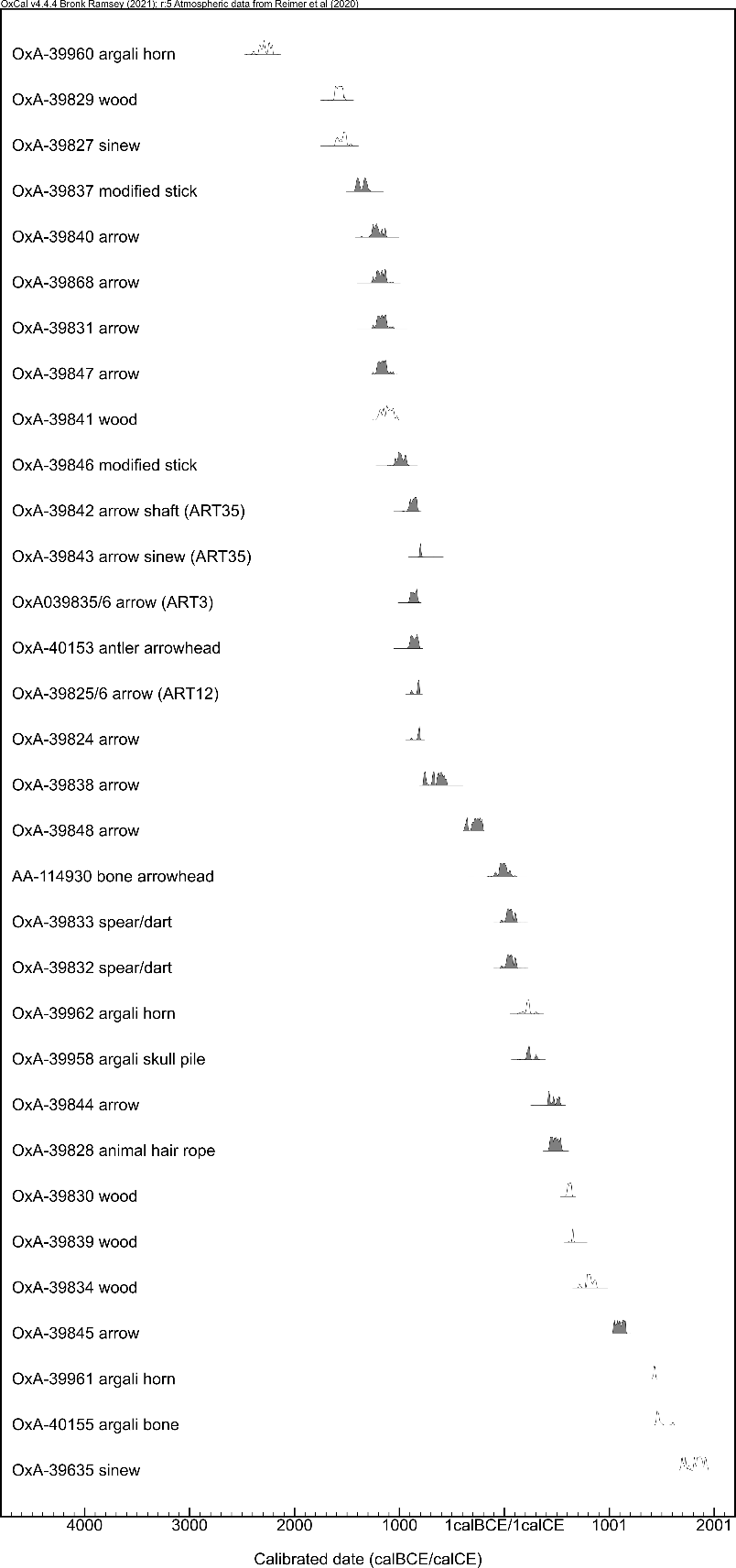
*

Supplementary Figure 6. Radiocarbon-dated artifacts and ecofacts at Tsengel Khairkhan and Ice Patch 3, showing near-continuous use of high mountain regions since at least ca. 2000 BCE. Dark shading indicates a clear cultural origin.


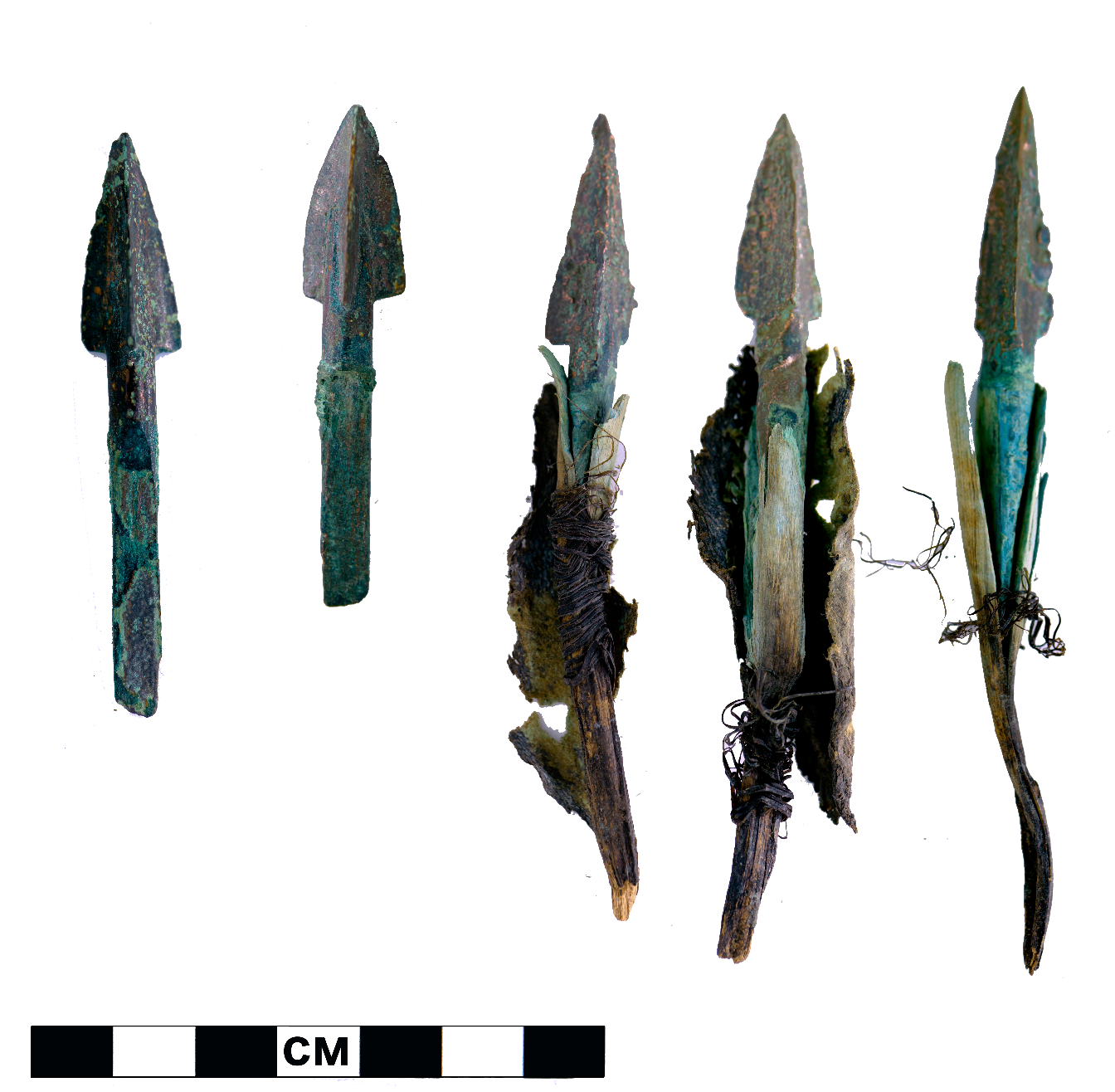


Supplementary Figure 7. Tripartite bronze arrowheads from Jargalantyn Am, Mongolia, dated to the early mid-first millennium BCE (Bayarsaikhan et al. 2020). Image: Jamsranjav Bayarsaikhan.


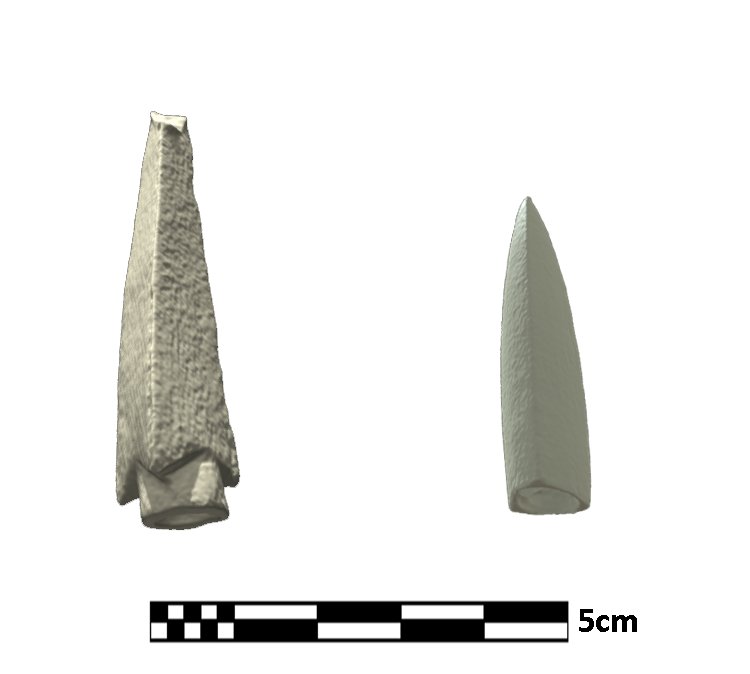


Supplementary Figure 8. 3D model showing antler arrowheads Artifact 13 (left), dating to the first millennium BCE, and Artifact 37 (right), dating to the early second millennium CE. Both projectiles were designed using a “friction fit” over a wooden arrow shaft. Image: William Taylor.

Table S1. Wooden artifacts identified during 2019 fieldwork, along with related radiocarbon dates and taxonomic identifications.

| **Site** | **ID** | **Description** | **^14^C YBP** | **Error (±)** | **Lab number (OxA)** | **Material** |
| --- | --- | --- | --- | --- | --- | --- |
| Ice Patch 3 (Khultsuut) | Artifact 20 | Wood piece | 3317 | 19 | 39829 | Unidentified |
| Tsengel Khairkhan | Artifact 30 | Large worked stick/possible "scare stick" | 3098 | 21 | 39837 | Buckthorn |
| Tsengel Khairkhan | Artifact 33a | Arrow shaft fragment | 2985 | 19 | 39840 | Willow |
| Tsengel Khairkhan | Artifact 9 | Arrow shaft fragment with feather fragments | 2966 | 19 | 39868 | Willow |
| Tsengel Khairkhan | Artifact 25 | Arrow shaft | 2956 | 20 | 39831 | Willow |
| Tsengel Khairkhan | Artifact 7a | Arrow shaft | 2951 | 18 | 39847 | Willow |
| Tsengel Khairkhan | Artifact 34a | Large wood fragment | 2921 | 21 | 39841 | Willow |
| Tsengel Khairkhan | Artifact 39 | Large worked stick/possible "scare stick" | 2839 | 18 | 39846 | Willow |
| Tsengel Khairkhan | Artifact 3(duplicate date 1 of 2) | Arrow shaft fragment | 2739 | 20 | 39835 | Willow |
| Tsengel Khairkhan | Artifact 35a | Complete wooden shaft with sinew, glue, ochre near fletching | 2736 | 18 | 39842 | Willow |
| Tsengel Khairkhan | Artifact 3 (duplicate date 2 of 2) | Arrow shaft fragment | 2712 | 18 | 39836 | Willow |
| Tsengel Khairkhan | Artifact 12 (duplicate date 1 of 2) | Arrow shaft fragment with nock end(collected ca. 2009) | 2689 | 18 | 39826 | Willow |
| Tsengel Khairkhan | Artifact 12(duplicate date 2 of 2) | Arrow shaft fragment with nock end (collected ca. 2009) | 2678 | 18 | 39825 | Willow |
| Tsengel Khairkhan | Artifact 11a | Arrow shaft fragment | 2666 | 18 | 39824 | Willow |
| Tsengel Khairkhan | Artifact 31 | Arrow shaft fragment | 2510 | 18 | 39838 | Willow |
| Tsengel Khairkhan | Artifact 8 | Willow arrow shaft with nock end | 2230 | 18 | 39848 | Willow |
| Tsengel Khairkhan | Artifact 28 | Possible atlatl or dart shaft fragment | 1967 | 18 | 39833 | Willow |
| Tsengel Khairkhan | Artifact 27 | Possible atlatl or dart, with scarf joint | 1965 | 19 | 39832 | Willow |
| Tsengel Khairkhan | Artifact 36 | Arrow shaft fragment | 1621 | 17 | 39844 | Willow |
| Tsengel Khairkhan | Artifact 21 | Wood piece | 1448 | 17 | 39830 | Unidentified |
| Tsengel Khairkhan | Artifact 32a | Wood piece | 1383 | 17 | 39839 | Willow |
| Tsengel Khairkhan | Artifact 29 | Wood piece | 1221 | 17 | 39834 | Willow |
| Tsengel Khairkhan | Artifact 37a | Arrow shaft fragment | 938 | 17 | 39845 | Willow |
| Tsengel Khairkhan | Artifact 1 | Large worked stick/possible "scare stick" | 1.25058 (modern) | 0.00244 | 39823 | Elm |
| Tsengel Khairkhan | Artifact 40 | Willow shaft fragment | undated | | | Willow |
| Tsengel Khairkhan | Artifact 37e | Arrow shaft fragment | undated | | | Willow |
| Tsengel Khairkhan | Artifact 37d | Arrow shaft fragment | undated | | | Willow |
| Tsengel Khairkhan | Artifact 37c | Arrow shaft fragment | undated | | | Willow |
| Tsengel Khairkhan | Artifact 37b | Arrow shaft fragment | undated | | | Willow |
| Tsengel Khairkhan | Artifact 34b | Large wood fragment | undated | | | Willow |
| Tsengel Khairkhan | Artifact 33b | Arrow shaft fragment | undated | | | Willow |
| Tsengel Khairkhan | Artifact 32c | Wood piece | undated | | | Willow |
| Tsengel Khairkhan | Artifact 32b | Wood piece | undated | | | Willow |
| Tsengel Khairkhan | Artifact 29 | Pointed arrow shaft | undated | | | Willow |
| Tsengel Khairkhan | Artifact 24 | Arrow shaft fragment | undated | | | Willow |
| Tsengel Khairkhan | Artifact 23 | Arrow shaft fragment | undated | | | Willow |
| Ice Patch 3 (Khultsuut) | Artifact 19 | Wood piece | undated | | | Unidentified |
| Tsengel Khairkhan | Artifact 11b | Arrow shaft fragment | undated | | | Willow |
| Tsengel Khairkhan | Artifact 10 | Wood piece | undated | | | Willow |

Table S2. Bone, antler, sinew, and hair artifacts identified during 2019 fieldwork, along with related radiocarbon dates and taxonomic identifications.

| **Site** | **ID** | **Description** | **^14^C YBP** | **Error (±)** | **Lab number** | **Material** |
| --- | --- | --- | --- | --- | --- | --- |
| Tsengel Khairkhan | Taphonomy survey tract, Sample 15 | Argali horn sheath fragment | 3840 | 13 | OxA-39960 | Argali horn |
| Tsengel Khairkhan | Taphonomy survey tract, Sample 4 | Argali bone fragment | 1828 | 20 | OxA-39962 | Argali bone |
| Tsengel Khairkhan | --- | Argali skull pile | 1816 | 20 | OxA-39958 | Argali horn |
| Tsengel Khairkhan | Taphonomy survey tract, Sample 39 | Argali horn sheath fragment | 487 | 19 | OxA-39961 | Argali horn |
| Tsengel Khairkhan | Taphonomy survey tract, Sample 1 | Argali horn sheath fragment | 407 | 22 | OxA-40155 | Argali bone |
| Tsengel Khairkhan | Artifact 14 | Sinew | 3275 | 28 | OxA-39827 | Sheep sinew |
| Tsengel Khairkhan | Artifact 35 | Sinew | 2625 | 19 | OxA-39843 | Deer sinew |
| Tsengel Khairkhan | Artifact 5 | Sinew | 131 | 20 | OxA-39635 | Non-mammalian sinew |
| Tsengel Khairkhan | Artifact 13 | Antler arrowhead | 2716 | 24 | OxA-40153 | Deer antler |
| Tsengel Khairkhan | Artifact 26 | Bone arrowhead | 2024 | 32 | AA-114930 | Sheep bone |
| Ice Patch 3 (Khultsuut) | Artifact 18 | Camel hair rope | 1582 | 18 | OxA-39828 | Camel hair |
| Ice Patch 1 (Khultsuut) | Bone 3 | Sheep/goat tooth | 1.21608 | 0.00308 | OxA-40154 | Ovicaprid tooth |

Table S3. ZooMS results for artifacts from Tsengel Khairkhan. Species identifications are provided first based on collagen only, with contextual identifications based on material type in parentheses for antler artifacts. Diagnostic markers for Ovis and Deer/Saiga/Gazelle are found in column C.

| **Specimen ID** | **Identification (ZooMS** | **P1** | **A** | **A'** | **B** | **C** | **P2** | **D** | **E** | **F** | **F'** | **G** | **G'** |
| --- | --- | --- | --- | --- | --- | --- | --- | --- | --- | --- | --- | --- | --- |
| Artifact 38 (antler) | *Deer/saiga/gazelle* (*Cervus elaphus)* | 1105.6 | 1180.6 | 1196.6 | 1427.7 | 1550.8 | 1648.8 | 2131.1 | 2792.3 | 2883.4 | 2899.4 | 3017.5 | 3033.5 |
| Artifact 13 (antler) | *Deer/saiga/gazelle* (*Cervus elaphus)* | 1105.6 | 1180.6 | 1196.6 | 1427.7 | 1550.8 | 1648.8 | 2131.1 | 2792.3 | 2883.4 | 2899.4 | 3017.5 | 3033.5 |
| Artifact 26 (bone) | *Ovis* | 1105.6 | 1180.6 | 1196.6 | 1427.7 | 1580.8 | 1648.8 | 2131.1 | 2792.3 | 2883.4 | 2899.4 | 3017.5 | 3033.5 |
| Artifact 35 (sinew) | *Deer/saiga/gazelle* | 1105.6 | 1180.6 | 1196.6 | 1427.7 | 1550.8 | 1648.8 | 2131.1 | 2792.3 | 2883.4 | 2899.4 | 3017.5 | 3033.5 |
| Artifact 5 (sinew) | *Non mammalian* |  |  |  |  |  |  |  |  |  |  |  |  |
| Artifact 14 (sinew) | *Ovis* | 1105.6 | 1180.6 | 1196.6 | 1427.7 | 1580.8 | 1648.8 | 2131.1 | 2792.3 | 2883.4 | 2899.4 | 3017.5 | 3033.5 |
| Bone 3 (Tooth) | *Ovis* | 1105.6 | 1180.6 | 1196.6 | 1427.7 | 1580.8 | 1648.8 | 2131.1 | 2792.3 | 2883.4 | 2899.4 | 3017.5 | 3033.5 |

**Supplementary Appendix A. Animal hair microscopy and species identification.**

*Methods*

The sample was analyzed using transmitted light microscopy (TLM) and scanning electron microscopy (SEM) in order to examine both internal and external microstructures. Results were compared with fibers in the investigator’s reference collection, including sheep (*Ovis aries*), goat (*Capra aegagrus hircus*), cattle (*Bos taurus*), horse (*Equus caballus*) and Bactrian camel (*Camelus bactrianus*) and with published reference images [1-3].

SEM analysis was conducted at the Center for Nanoscale Systems (CNS) at Harvard University using a Zeiss Ultra55 FESEM. The specimen was mounted with carbon tape on an aluminum stub and sputter coated with platinum/palladium (Pt/Pd (80:20) to a thickness of 20 nm. The SEM was operated at an accelerating voltage of 15 Kv with a working distance of 10-12 mm.

*Results*

Tips and at least one root were present in the sample, suggesting that the fibers had been harvested through plucking or combing rather than shearing. Fine fibers were unmedullated, whereas intermediate and coarse fibers showed continuous, amorphous medulli with relatively straight margins and a diameter of about 1/3rd the total diameter of the fiber. The scales of fine fibers were in some places diagonal to the axis of the fiber, a feature diagnostic of camel *(Camelus bactrianus*) [1]. Coarse fibers showed a scale pattern of fine, transversal waves with rippled margins, also consistent with an identification of camel.

Given significant overlap in the fiber morphologies of some species and the potentially degraded quality of the scales in any archaeological fiber sample, a potential alternative identification of horse (i)—which was suggested by knowledgeable local informants upon observation of the whole artifact—has not been ruled out. Horse hair has a similar amorphous medullary structure and cuticular morphology of rippled waves, though in horses the scale margins tend to be smoother and the scales more pronounced in comparison with camel.

**References cited**

1. Rast-Eicher, A. Fibres: Microscopy of Archaeological Textiles and Furs. (Archaeolingua Alapítvány, 2016).

2.De Marinis, A. M. & Asprea, A. Hair identification key of wild and domestic ungulates from southern Europe. wbio 12, 305–320 (2006).

3.Appleyard, H. M. Guide to the Identification of Animal Fibres. (Wool Industries Research Association, 1960).

*





*

Supplementary Figure 9. SEM micrograph of animal-hair artifact from Khultsuut (top, 800x) as compared to camel (center, 377x) and horse (below, 300x) reference samples.

**Supplementary Appendix B. Paleoclimate summary for western Mongolia and adjoining regions.**

In contrast to the well-documented glacial history of the late Pleistocene [1-3], limited geomorphological evidence exists for glacier distribution between the early Holocene and the mid-Holocene [4], a generally wet and warm period referred to as the “Holocene climate optimum.” On the heels of this optimum, ice core data from the central Mongolian Altai (Tsambagarav Mountain) suggest initiation of glacial buildup around 4000 BCE [5]. By ca. 3000 BCE, a time period associated with the arrival of the region’s first pastoralists, glacial records suggest that the area’s climate became dry, but glaciation prevailed under cold conditions [5]. Between ca. 3500 and 1600 BCE, neoglacial advances occurred in the northern Altai, followed by warmer conditions until ca. 800 CE (Mongun-Taiga massif [6]). Similar neoglacial advances were reported in this region by Agatova et al. at North Chuya between ca. 2900-2200 BCE and again from 300 BCE – 300 CE [7,8]. In the western Altai (Belucha peak), glaciers persisted across the mid-Holocene [9], with periods of advances reported at ca. 4000 BCE, 2700-2000 BCE, 0CE – 500 CE, and 1700-1900 CE [10]. Across the Altai, the Little Ice Age (LIA) appears to have produced fresh, distinct moraines that serve as a proxy for maximum glacial extent during this period between the 13^th^ and 19^th^ centuries CE [5,8, 11-12]. At Tsengel Khairkhan, organic materials dating to the LIA are found in snow patches between the contemporary ice margin and the terminal moraines(Supplementary Figure 4).

Like glacial data, other paleoclimate proxies also provide useful data with relatively poor chronological resolution for understanding human use of high altitudes in the Altai since the Bronze Age. In the Russian Altai, various datasets suggest warm periods between 1300-250 BCE and again between 300-1200 CE (dendrochronology) or ca. 650-1100 CE (ice cores), bracketed by cooler periods. These intervals were followed by the onset of the LIA at ca. 1500-1600 CE [7,9,13]. In the southern (Chinese) Altai, Feng et al. [14] reported warm and wet conditions between ca. 2000 BCE – 800 CE, followed by cool and dry conditions. Closer to Tsengel Khairkhan, at the site of Achit Nuur to the north in western Mongolia, Sun et al. [15] identified warm and wet conditions between ca. 3000 BCE – 400 CE, followed by a drier period. In the Uvs Nuur basin, to the northeast, wetter conditions prevailed prior to ca. 1000-500 BCE [16,17].

The most precise insights into the region’s paleoclimate come from the immediate vicinity of the study area from Khoton Nuur and Dayan Nuur, a pair of large glacial lakes along Tsengel Khairkhan’s southwestern margin. Based on analysis of lake sediments, Rudaya et al. [18] and Rudaya and Li [19] identified a local decrease in precipitation before ca. 2000 BCE, with an onset of wetter conditions after ca. 2000-1000 BCE. Palynological and macro-charcoal analysis from a peat bog south of nearby Dayan Nuur, suggested a comparatively moist and warm climate, similar to present conditions between ca. 1900 and 600 BCE, with generally colder, drier conditions between 600 BCE and 1450 CE [20]. Perhaps most importantly, Unkelbach and colleagues identify evidence of fire events – linked to warm, dry conditions – at ca. 500 BCE, ca. 150 CE, ca. 450 CE, and between ca. 1350-1450 CE [20].

**References cited**

1. Blomdin, R. et al. Glacial geomorphology of the Altai and Western Sayan Mountains, Central Asia. Journal of Maps vol. 12 123–136 (2016).
2. Klinge, M. et al. Late Pleistocene lake level, glaciation and climate change in the Mongolian Altai deduced from sedimentological and palynological archives. Quat. Res. 1–22.
3. Lehmkuhl, F., Klinge, M., Rother, H. & Hülle, D. Distribution and timing of Holocene and late Pleistocene glacier fluctuations in western Mongolia. Ann. Glaciol. 57, 169–178 (2016).
4. Solomina, O. N. et al. Holocene glacier fluctuations. Quat. Sci. Rev. 111, 9–34 (2015).
5. Herren, P.-A. et al. The onset of Neoglaciation 6000 years ago in western Mongolia revealed by an ice core from the Tsambagarav mountain range. Quat. Sci. Rev. 69, 59–68 (2013).
6. Ganyushkin, D. et al. Palaeoclimate, glacier and treeline reconstruction based on geomorphic evidences in the Mongun-Taiga massif (south-eastern Russian Altai) during the Late Pleistocene and Holocene. Quaternary International vol. 470 26–37 (2018).
7. Agatova, A. R. et al. Glacier dynamics, palaeohydrological changes and seismicity in southeastern Altai (Russia) and their influence on human occupation during the last 3000 years. Quaternary International vol. 324 6–19 (2014).
8. Agatova, A. R., Nazarov, A. N., Nepop, R. K. & Rodnight, H. Holocene glacier fluctuations and climate changes in the southeastern part of the Russian Altai (South Siberia) based on a radiocarbon chronology. Quat. Sci. Rev. 43, 74–93 (2012).
9. Aizen, E. M. et al. Abrupt and moderate climate changes in the mid-latitudes of Asia during the Holocene. J. Glaciol. 62, 411–439 (2016).
10. Serebryanny, L. R. & Solomina, O. N. Glaciers and Climate of the Mountains of the Former USSR during the Neoglacial. Mt. Res. Dev. 16, 157–166 (1996).
11. Lehmkuhl, F. Holocene glaciers in the Mongolian Altai: An example from the Turgen–Kharkhiraa Mountains. J. Asian Earth Sci. 52, 12–20 (2012).
12. Ganiushkin, D., Chistyakov, K. & Kunaeva, E. Fluctuation of glaciers in the southeast Russian Altai and northwest Mongolia Mountains since the Little Ice Age maximum. Environ. Earth Sci. 74, 1883–1904 (2015).
13. Schlütz, F. & Lehmkuhl, F. Climatic change in the Russian Altai, southern Siberia, based on palynological and geomorphological results, with implications for climatic teleconnections and human history since the middle Holocene. Veg. Hist. Archaeobot. 16, 101–118 (2007).
14. Feng, Z. et al. Vegetation changes and associated climatic changes in the southern Altai Mountains within China during the Holocene. Holocene 27, 683–693 (2017).
15. Sun, A., Feng, Z., Ran, M. & Zhang, C. Pollen-recorded bioclimatic variations of the last ∼22,600 years retrieved from Achit Nuur core in the western Mongolian Plateau. Quaternary International vol. 311 36–43 (2013).
16. Tian, F. et al. A modern pollen-climate calibration set from central-western Mongolia and its application to a late glacial-Holocene record. Journal of Biogeography vol. 41 1909–1922 (2014).
17. Naumann, S. Spät-und postglaziale Landschaftsentwicklung im Bajan Nuur Seebecken (Nordwestmongolei). Erde (1999).
18. N. Rudaya, et al. Holocene environments and climate in the Mongolian Altai reconstructed from the Hoton-Nur pollen and diatom records: a step towards better understanding climate
19. N. Rudaya, & H-C. Li, A new approach for reconstruction of the Holocene climate in the Mongolian Altai: The high-resolution δ13C records of TOC and pollen complexes in Hoton-Nur Lake sediments. J. Asian Earth Sci. 69, 185–195 (2013).
20. Unkelbach, J. et al. Late Holocene (Meghalayan) palaeoenvironmental evolution inferred from multi-proxy-studies of lacustrine sediments from the Dayan Nuur region of Mongolia. Palaeogeography, Palaeoclimatology, Palaeoecology vol. 530 1–14 (2019).

*
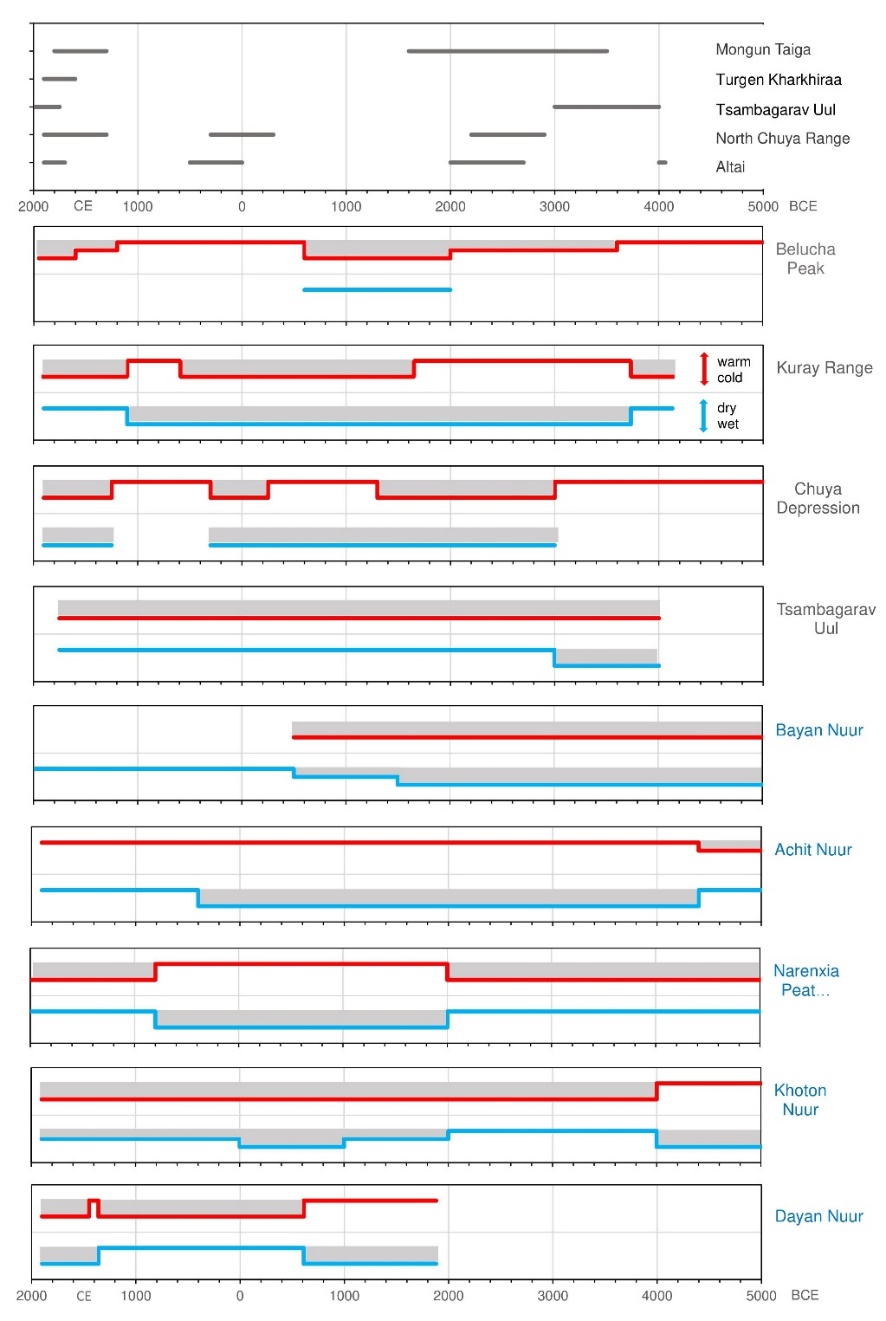
*

Supplementary Figure 10. Relative climate changes and reconstructed glacier advances based on different archives from literature sources. Black labelled regions indicate data from mountain areas, blue labels indicate data from basins and valleys, and site locations are labeled in Figure 1 (main text). Red line = temperature; blue line = moisture; grey beams indicate periods favoring glacier ice development; black line = Glacier advances. Due to the arid climate in the Mongolian Altai, glaciation may be restricted by both temperature and precipitation, with decreasing temperature reducing ablation, and increasing precipitation leading to a positive glacier balance. If both factors occur together, then glacier advance occurs.
